# Balancing Anti‐Inflammation and Neurorepair: The Role of Mineralocorticoid Receptor in Regulating Microglial Phenotype Switching After Traumatic Brain Injury

**DOI:** 10.1111/cns.70404

**Published:** 2025-04-25

**Authors:** Bin Zhang, Miao Bai, Mengshi Yang, Yumei Wang, Xueling Zhang, Xiyu Chen, Min Gao, Baiyun Liu, Guangzhi Shi

**Affiliations:** ^1^ Department of Critical Care Medicine, Beijing Tiantan Hospital Capital Medical University Beijing China; ^2^ Department of Neurology The First Hospital of Tsinghua University Beijing China; ^3^ Department of Animal Laboratory Beijing Neurosurgical Institute Beijing China; ^4^ Department of Neurosurgery, Beijing Tiantan Hospital Capital Medical University Beijing China

**Keywords:** fludrocortisone, mineralocorticoid receptor microglia polarization, neuroinflammation, traumatic brain injury

## Abstract

**Background:**

As potent anti‐inflammatory agents, glucocorticoids (GCs) have been widely used in the treatment of traumatic brain injury (TBI). However, their use remains controversial. Our previous study indicated that although dexamethasone (DEX) exerted anti‐inflammatory effects and protected the blood–brain barrier (BBB) by activating the glucocorticoid receptor (GR) after TBI, it also impeded tissue repair processes due to excessive anti‐inflammation. Conversely, fludrocortisone, acting as a specific mineralocorticoid receptor (MR) agonist, has shown potential in controlling neuroinflammation and promoting neurorepair, but the underlying mechanisms need further exploration.

**Objective:**

This study aimed to explore the impact of the MR agonist fludrocortisone on microglia polarization, angiogenesis, functional rehabilitation, and associated mechanisms after TBI.

**Methods:**

We established a mice controlled cortical impact model, and then immunofluorescence staining, western blot, rt‐PCR, and MRI were performed to investigate microglia polarization, angiogenesis, and brain edema in the ipsilateral hemisphere after TBI and fludrocortisone treatment. Subsequently, functional tests including morris water maze, sucrose preference test, and forced swimming test were conducted to evaluate the effects of fludrocortisone treatment on neurofunction after TBI.

**Results:**

Our results revealed that fludrocortisone suppressed neuroinflammation, enhanced angiogenesis and neuronal survival, and promoted functional rehabilitation by inducing a shift in microglia phenotype from M1 to M2 via the JAK/STAT6/PPARγ pathway. Additionally, the PI3K/Akt/HIF‐1α pathway was involved in VEGF expression and in the process of angiogenesis.

**Conclusion:**

Fludrocortisone, the specific MR agonist, exerted anti‐neuroinflammatory and neuroprotective effects by regulating phenotypic switching of microglia from M1 to M2 rather than suppressing all types of microglia. Our study provided a theoretical basis for the therapeutic strategy of GCs targeting neuroinflammation after TBI.

## Graphical abstract

Instead of suppressing all types of microglia, activation of MR by FLUDRO exerts anti‐neuroinflammatory and neuroprotective effects by promoting the transformation of M1‐like microglia to M2‐like microglia via the JAK/STAT6/PPARγ pathway, promoting VEGF expression and angiogenesis via the PI3K/Akt/HIF‐1α pathway, increasing the number of survival neurons, and improving functional rehabilitation after TBI.

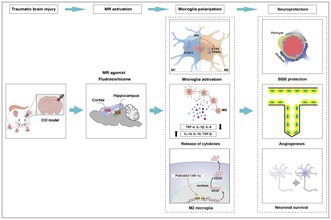

## 1 Introduction

Among the secondary injury mechanisms, neuroinflammatory responses can occur throughout the entire course of traumatic brain injury (TBI) and interact with almost all other secondary injury mechanisms [1]. Interestingly, neuroinflammation plays dual and even opposing roles in the injury processes. On the one hand, immune cells recruited by damaged neuronal tissue can exacerbate brain damage through the release of neurotoxic substances; on the other hand, they also facilitate brain tissue repair, including angiogenesis and neurogenesis by clearing cell debris, inhibiting local inflammation, and secreting trophic factors [2, 3, 4]. Microglia, the primary innate immune cells in the brain, constitute approximately 10% of total brain cells [5]. After TBI, these cells are rapidly activated by damage‐associated molecular patterns (DAMPs) and exert both neurotoxic and neuroprotective effects depending on their phenotypes: pro‐inflammatory (M1) or anti‐inflammatory (M2). The M2 phenotype has been found to be the most potent modulators of CNS repair and regeneration by releasing proangiogenic factors, such as vascular endothelial growth factor (VEGF) and play important roles in angiogenesis. The distinct phenotypes of activated microglia and macrophages can either support or hinder neurological recovery after stroke or other injuries [6, 7, 8, 9], and numerous signaling pathways are involved in microglial polarization [10, 11]. Therefore, clarifying the interplays between these pathways and developing therapeutic strategies that target microglial phenotype shifts, rather than a blanket suppression of microglia, is crucial for TBI management.

A multitude of experimental and clinical studies have explored the use of anti‐inflammatory agents to improve outcomes in TBI management [12, 13]. As potent anti‐inflammatory agents, high doses of glucocorticoids (GCs) such as dexamethasone (DEX) and methylprednisolone (MP) have been widely used for decades in brain tumors and other neurosurgical interventions to alleviate edema by inhibiting inflammation and stabilizing vascular permeability [14, 15, 16]. However, their efficacy in TBI patients remains uncertain and controversial [17]. Therefore, whether the anti‐inflammatory effects of GCs are beneficial or detrimental after TBI may be revisited in the future.

In the brain, GCs act through two types of receptors: the glucocorticoid receptor (GR) and the mineralocorticoid receptor (MR), which are not uniformly distributed. MR has a higher affinity for natural GCs like cortisol/corticosterone (CORT) compared to GR and is extensively activated under basal CORT levels, while GR activation occurs primarily during ultradian peaks or stress [18]. The special activation patterns of the two receptors are vital for regulating neuroinflammation, stress, and neuronal survival, whereas the imbalance of MR/GR is associated with various neurodegenerative and mental disorders [19, 20]. Our previous study indicated that the excessive GR activation by DEX exacerbated corticosterone insufficiency, inhibited angiogenesis, and impaired neurofunctional recovery after TBI, whereas MR activation by fludrocortisone (FLU) or CORT mitigated DEX‐induced impairments by promoting a rebalancing of brain corticosteroid receptors [21, 22, 23].

Both MR and GR are highly expressed in microglia, making them direct targets for GCs. While the anti‐inflammatory effects of GCs are primarily mediated by GR, which suppresses most activated microglia [24, 25], MR has been reported to have both anti‐inflammatory and pro‐inflammatory effects in the brain [26, 27]. The roles of MR in regulating microglial phenotype switching post‐TBI, however, remain unexplored. This study hypothesizes that MR is a pivotal modulator of microglial M1/M2 polarization in the acute phase of TBI. We propose that the MR‐specific agonist FLU can inhibit neuroinflammation and enhance brain tissue repair by promoting the transition of microglia from the M1 to M2 phenotype. To test this hypothesis, we utilized an experimental TBI model in mice treated with FLU.

## 2 Material and Methods

### 2.1 Animals and Controlled Cortical Impact (CCI) Model

All experimental procedures were approved by the Capital Medical University Institutional Animal Care and Use Committee. Adult male C57BL/6J mice, purchased from Beijing Vital River Experimental Animals Technology Ltd., were used in the present study. All mice were provided free access to food and water and housed individually under controlled temperature (22°C) and humidity (60%). All animals were habituated to the animal facility conditions for 7 days before the beginning of the experiments.

The CCI brain injury model was conducted with a PCI3000 PinPoint Precision Cortical Impactor (Hatteras Instruments, Cary, NC, USA) as we previously described [22]. Briefly, mice were anesthetized by isoflurane inhalation and fixed in a stereotaxic frame (RWD Life Science Co., Shenzhen, Guangdong, China). After the scalp was incised and the skull was fully exposed, a craniotomy (approximately 4 mm in diameter) was performed in the middle of the right parietal bone, keeping the underlying dura intact. At last, a severe TBI model was induced Using the impact parameters described previously (velocity: 3.5 m/s; compression time: 400 ms; and depth: 2 mm) with a 3‐mm circular impact tip. Throughout the surgical procedures, a thermal pad was used to maintain the body temperature of the mouse at about 37.0°C ± 0.5°C.

### 2.2 Experimental Groups and Treatments

#### 2.2.1 Experiment 1

Based on whether they underwent CCI and the subsequent treatments, mice were assigned to five distinct groups: 1) sham + dimethyl sulfoxide (DMSO) control group (sham); 2) CCI + DMSO group (CCI); 3) TBI + FLU (1 mg/kg, for 3 days after CCI, F6127, Sigma) group (FLU); 4) CCI + BEVA (10 mg/kg after CCI, Avastin, 25 mg/mL; Roche, Switzerland) (CCI + BEVA); and 5) TBI + FLU + BEVA (FLU + BEVA). FLU was dissolved using DMSO with a final dilution of < 1%, and BEVA was dissolved in sodium chloride. The mice were treated with intraperitoneal injections for 7 days. All the drugs' dosages were chosen based on pilot experiments from our laboratory and previous studies from the literature [28, 29].

#### 2.2.2 Experiment 2

To investigate the effect of microglia on angiogenesis, CSF‐1R antagonist PLX5622 was continuously administered to induce widespread loss of microglia. Mice were divided into five groups: (1) sham group; (2) CCI group; (3) FLU (1 mg/kg, F6127, Sigma) group; (4) CCI + PLX5622 group; and (5) CCI + FLU + PLX5622 (1 mg/kg, F6127, Sigma) group (FLU + PLX5622). Vehicle or PLX5622 diet (at a concentration of 1200 mg/kg, Plexxikon Inc) was provided to feed mice for 3 weeks to deplete microglia before TBI. Mice were maintained on the experimental diets for the duration of the study. This dose and time can induce a widespread loss of microglia, including in the hippocampus throughout the experimental time periods after TBI [30].

#### 2.2.3 Experiment 3

For the downstream signal pathway mechanism study, mice were injected with PI3K inhibitor LY294002 via the lateral ventricle and divided into five groups: (1) sham group; (2) CCI group; (3) FLU (1 mg/kg, F6127, Sigma) group; (4) CCI + LY294002 group; (5) TBI + FLU + LY294002 group (FLU + LY294002). LY294002 was dissolved in 0.1% DMSO with normal saline at a concentration of 10 mM, and 2 μL of LY294002 solution was injected into the lateral ventricle of mice 30 min before CCI, following the stereotaxic coordinates: 1.0 mm lateral, 2.5 mm depth, and − 0.25 mm AP to bregma [6]. The injection was performed at a rate of 0.5 μL/min, and the needle stayed in place for 5 min after injection of the drugs, followed by slow removal.

#### 2.2.4 ELISA Assay

Inflammatory factors and cytokines (i.e., tumor necrosis factor‐alpha (TNF‐α), interleukin‐6 (IL‐6), interleukin‐1(IL‐1), interleukin‐4 (IL‐4), interleukin‐10 (IL‐10), transforming growth factor‐beta (TGF‐β), and interleukin‐4 (IL‐4) and VEGF) in the ipsilateral hemisphere were also detected. Tissue samples from the ipsilateral hemisphere were collected at 1, 3, and 7 days post‐injury. After homogenization of the samples, supernatants were obtained and used for further analysis. Corticosterone (CORT) and cytokine levels were measured using specific enzyme‐linked immunosorbent assay (ELISA) kits (Nanjing KeyGen Biotech. Co. Ltd.) according to the manufacturer's instructions.

### 2.3 Immunofluorescence Staining

Mice were sacrificed at different time points after TBI. The brains were removed, and brain paraffin slices were prepared for immunofluorescence staining according to our previous procedures [22]. Brain slices were single‐stained (CD31, postinjury day 1, 3 and 7; NEUN, postinjury day 7) or double‐stained (CD16/32 + Iba‐1 and CD206 + Iba‐1, postinjury day 3) for immunohistochemical evaluation. Briefly, the brain slice and cells were incubated with primary antibodies overnight at 4°C. Alexa Fluor 488 (1:500, ab150113, Abcam, UK) or 594 (1:500, ab150080, Abcam, UK)—conjugated secondary antibodies were then added and incubated for 1 h at room temperature. Antibodies are listed as follows: anti‐NeuN (1:200, Abcam, ab177487, UK), CD31 (1:200, Abcam, ab222783, UK), anti‐CD16/32 antibody (1:500, Abcam, ab223200, UK), anti‐CD206 antibody (1:400, Abcam ab64693, UK), anti‐Iba‐1 antibody (1:200, Abcam, ab178846, UK) overnight at 4°C. The slices were then washed with PBS and incubated with Alexa Fluor 647‐conjugated donkey anti‐rabbit IgG and Alexa Fluor 488‐conjugated goat anti‐mouse IgG at room temperature for 2 h. Finally, the samples were counterstained with DAPI (Sigma‐Aldrich, St. Louis, MO) for 10 min. Digital images of the whole brain sections were obtained by a MIDI FL (3D Histech, Hungary) system.

The quantifications of angiogenesis, microglia, and neurons in the ipsilateral cortex and hippocampus were conducted as previously described [23]. Five equally sized, non‐overlapping zones in the perilesional cortex or the ipsilateral hippocampus were acquired for cell counts. For neuron counts in the ipsilateral hippocampus, three non‐overlapping zones in the ipsilateral CA3 and CA1 and six zones in the ipsilateral dentate gyrus were used. Data are presented as the number of positive cells per mm^2^ and the number of hippocampal neurons per mm.

### 2.4 Determination of Brain Water Content

To assess brain water content, we measured the wet and dry weights of the brain tissue on post‐injury days 1, 3, and 7. The brain was extracted from the mice without prior transcardial perfusion, and the injured cerebral hemisphere was carefully dissected and weighed to obtain the wet weight. The tissue was then placed in an oven at 80°C for 72 h to dry until a constant dry weight was achieved. The brain water content was determined using the formula: brain water content (%) = (wet weight‐dry weight)/wet weight×100%.

### 2.5 Evans Blue Extravasation

To evaluate the permeability of the blood–brain barrier (BBB), we quantified Evans blue (EB) extravasation into the injured hemisphere on post‐injury days 1, 3, and 7. Mice were injected with a 4% EB solution (3 mL/kg) via the tail vein. After a period of 4 h, the animals were anesthetized and transcardially perfused with PBS to remove circulating EB. The injured cerebral hemisphere was then dissected, weighed, and homogenized in formamide at 72°C for 3 days to ensure complete extraction of EB. Following centrifugation, the supernatant was collected, and its absorbance was measured at 620 nm using a spectrophotometer (Bio‐Rad, Hercules, CA, United States).

### 2.6 Magnetic Resonance Imaging (MRI)

In this study, an animal MR scanner (Bruker 7.0 T, Germany) was used to assess the volume of brain edema on post‐injury day1, 3, and 7. According to previously described protocols, all animals were fully anesthetized by pentobarbital. Each group underwent axial T1/T2/ADC imaging with the following parameters: the FOV was 35 mm × 35 mm, and the layer thickness was 1 mm. MRI images were read and reviewed by RadiAnt DICOM viewer (version 4.6.9, Medixant, Poznan, Poland) and annotated by radiologists.

### 2.7 Western Blot Analysis

Total and nuclear protein extraction was conducted for each group (*n* = 6) as detailed in our previous work [20]. Chemiluminescence detection was performed using the Bio Spectrum 500 Imaging System (UVP Co., Upland, CA, USA). The primary antibodies used were as follows: anti‐MR (100 kDa) (1:100, ab64457, Abcam, UK), anti‐STAT 1(87 kDa) antibody (1:1000, ab92506, Abcam, UK), anti‐p‐STAT1 antibody (S727, 1:1000, ab109461, Abcam, UK), anti‐STAT6 (94 kDa) antibody (1:1000, ab32520, Abcam, UK), anti‐p‐STAT6 antibody (94 kDa) (1:500, ab263947, Abcam, UK), anti‐PPARγ (57 kDa) (1:500, Abcam, ab209350), rabbit anti‐AKT (56 kDa) (1:500; ab8805, Abcam, UK), anti‐P‐AKT (56 kDa) (ser 473, 1:5000; ab81283, Abcam, UK), anti‐Hif‐1α (105 kDa)(1:5000; ab179483, Abcam, UK), anti‐GAPDH (36 kDa) (1:5000, Abcam, ab8245), and anti‐Histone H3 (15 kDa) (1:2000; ab1791, Abcam, U). The protein blots were developed via chemiluminescence (Bio Spectrum 500 Imaging System; UVP Co., Upland, CA, USA). The relative band density was measured with ImageJ (version 1.49) and normalized to that of GAPDH, and the percent expression compared to that of sham controls was calculated for each sample.

### 2.8 Isolation of Microglia/Monocytes In Vivo From Sites of Injury

Microglia/macrophages were isolated from brain tissue using magnetic bead‐conjugated anti‐CD11b antibody according to previous procedures [7]. Briefly, ipsilateral cortex and hippocampus were collected from experimental mice and were dissociated with Adult Brain Dissociation Kit (Miltenyi Biotec, 130–093‐231, Germany). The dissociated brain samples were resuspended and passed through a cell Strainer (70 μm). After the debris, myelin, and red blood cells were removed, the cell sample was incubated with anti‐CD11b microbeads (Miltenyi Biotec, 130–049‐601, Germany). Finally, microglia/macrophages were isolated by MACS Separator (Miltenyi Biotec, 130–090‐976) according to the manufacturer's guidelines.

### 2.9 RNA Extraction and RT‐PCR

The total RNA from microglia isolated from the ipsilateral cortex and hippocampus at 72 h after TBI was extracted using TRIzol reagent (Invitrogen, Carlsbad, CA, USA), and cDNA was synthesized from 1 μg RNA using the Prime‐Script RT Reagent Kit (Thermo, USA). Next, the produced cDNA was amplified by quantitative real‐time PCR on a Quant Studio 5 System with SYBR Select Master Mix (Life Technologies, USA). β‐ACTIN was employed as the control for the target genes. The expression levels of the mRNA were then reported as fold changes versus sham controls. The sequences of the primer pairs for target genes are as shown below (Table 1):

**TABLE 1:** Primer sequences used in RT‐qPCR.

gene	Forward primer sequence (5′‐3′)	Reverse primer sequence (5′‐3′)
CD206	CTCTGTTCAGCTATTGGACGC	TGGCACTCCCAAACATAATTTGA
CD16	ATGTGGCAGCTACTACTACCA	ACCCACTTGGGGTCTAGGTTC
CD32	AATCCTGCCGTTCCTACTGATC	GTGTCACCGTGTCTTCCTTGAG
TGF‐β1	CCACCTGCAAGACCATCGAC	CTGGCGAGCCTTAGTTTGGAC
IL‐4	GGTCTCAACCCCCAGCTAGT	GCCGATGATCTCTCTCAAGTGAT
IL‐10	CCAAGCCTTATCGGAAATGA	TTTTCACAGGGGAGAAATCG
TNF‐α	CAGGCGGTGCCTATGTCTC	CGATCACCCCGAAGTTCAGTAG
IL‐6	CTGCAAGAGACTTCCATCCAG	AGTGGTATAGACAGGTCTGTTGG
IL‐1β	GCAACTGTTCCTGAACTCAACT	ATCTTTTGGGGTCCGTCAACT
MR	GAAAGGCGCTGGAGTCAAGT	CCATGTAGCTGTTCTCATTGGT
β‐Actin	GTGACGTTGACATCCGTAAAGA	GCCGGACTCATCGTACTCC

### 2.10 Behavioral Test

#### 2.10.1 Morris Water Maze (MWM) Assay

The morris water maze (MWM) test was performed to evaluate spatial learning ability as we described previously [21]. The probe trials were conducted pre‐ and post‐injury (3 and 7 days) to evaluate reference memory. Each mouse underwent 4 trials per day for 5 consecutive days (8–4 days before CCI) to find the platform submerged below the water. Twenty‐four hours after training, the hidden platform was removed, and a probe trial was conducted. The goal quadrant time and distance were recorded by a video tracking system.

#### 2.10.2 Sucrose Preference Test

Sucrose preference test (SPT) was performed to measure anhedonia, a typical symptom of depression‐like behavior after TBI. According to the procedures described previously [30], before the formal test, the mouse was raised in a single cage and was given two identical bottles containing 1% sucrose solution and drinking water for 12 h. After 24 h of food and water deprivation, SPT was conducted for 2 days as follows: each mouse was allowed free access to 1% sucrose solution and drinking water for 12 h; the two bottles were switched in the second 12 h. The weights of the two bottles were recorded before and after SPT, and the sucrose preference was calculated as follows: Sucrose preference (%) = sucrose consumption/total consumption × 100%.

#### 2.10.3 Forced Swimming Test

The forced swim test (FST) was conducted as previously described [30]. A mouse was put into a Perspex cylinder (30 cm in height and 18 cm in diameter) with 16 cm of water for 6 min (22°C ± 0.5°C). The duration of immobility and swimming in the water was counted, and the whole experimental process is recorded by the SMART 3.0 imaging system (Panlab, Germany).

### 2.11 Statistical Analysis

All data are presented as the means ± standard deviations (SDs). The data were analyzed using SPSS 22.0 (IBM Corporation, USA). All data conform to the normal distribution using the Shapiro–Wilk normality test. Repeated analysis of variance (ANOVA) was used to analyze latency time. The other results were analyzed by one‐way ANOVA followed by Tukey's post hoc test, and graphs were generated using the GraphPad Prism 9 software. A *p*‐value < 0.05 was considered statistically significant.

## 3 Results

### 3.1 FLU Treatment Improved Neurological Functions After TBI

**FIGURE 1:**
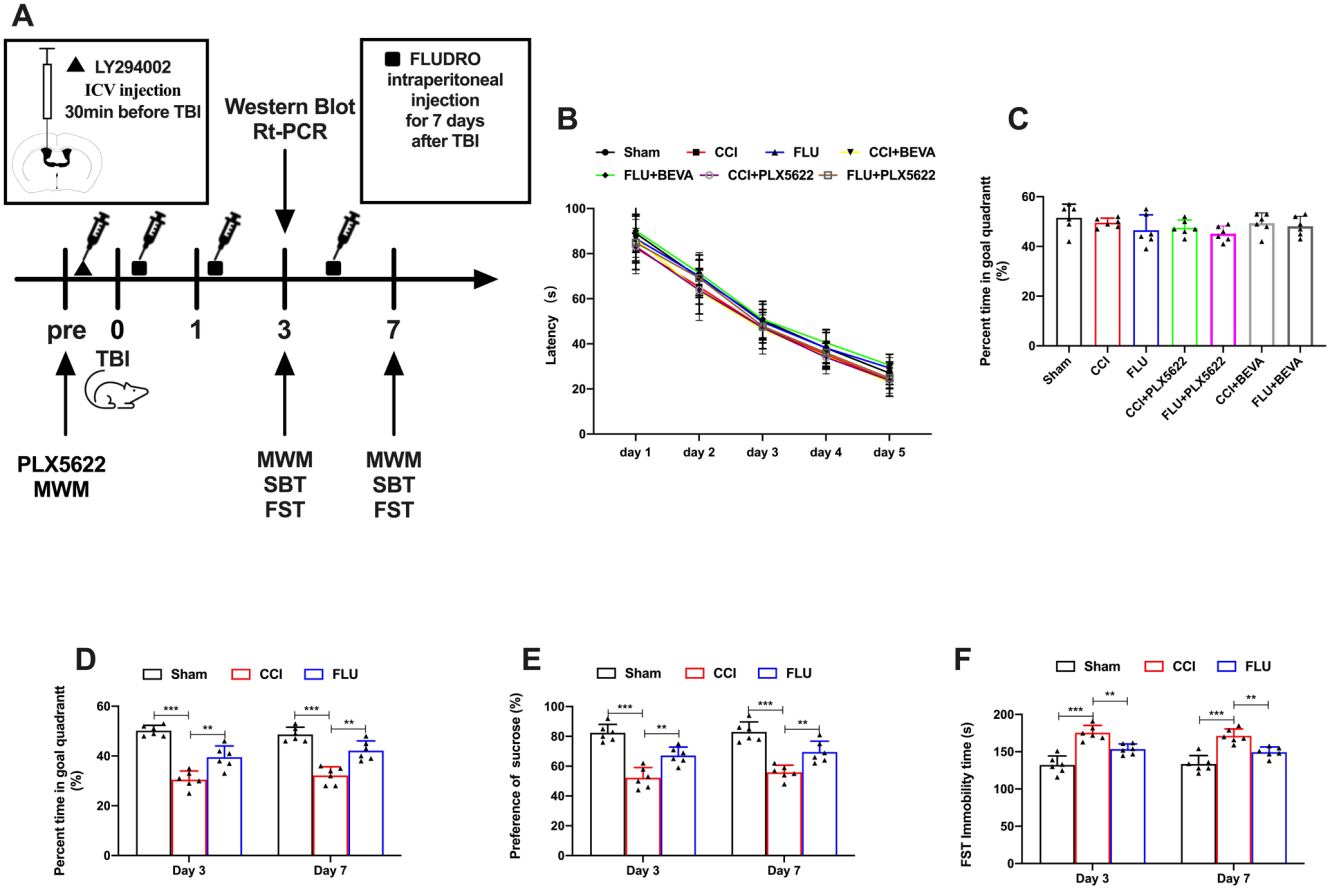
FLU treatment improved neurological functions after TBI. (A) Experimental designs and animal groups with different treatments and neurological function assessment.; (B, C) Quantification of the escape latency and the percentage of time in goal quadrant before CCI; (D) Quantification of the percentage of time on postinjury day 3 and 7; (E) Quantification of the preference of sucrose on postinjury day 3 and 7; (F) Quantification of the FST immobility time on postinjury day 3 and 7. *p < 0.05, **p < 0.01, ***p < 0.001. The data are presented as the means ± SD (*n* = 6).

In this study, we evaluated emotional and spatial memory capabilities using the MWM, FST, and SPT following TBI (Figure 1A). Prior to TBI, we assessed the baseline spatial learning and memory ability in all experimental groups by measuring latency time and conducting probe trials. The results showed no significant differences in latency time and the percentage of time spent in the goal quadrant across all groups before TBI (*p* > 0.05) (Figure 1B,C). However, there was a significant decrease in both the percentage of time in the goal quadrant and sucrose preference after TBI (*p* < 0.05) (Figure 1D,E). Additionally, the immobility time in the FST was significantly increased (*p* < 0.05) (Figure 1F). In contrast, treatment with FLU, a specific MR agonist, ameliorated all the aforementioned neurological deficits compared to the CCI group (*p* < 0.05). These results revealed that activation of MR by FLU exerted neuroprotective effects after TBI.

### 3.2 FLU Treatment Promoted Angiogenesis and Neuronal Survival After TBI

**FIGURE 2:**
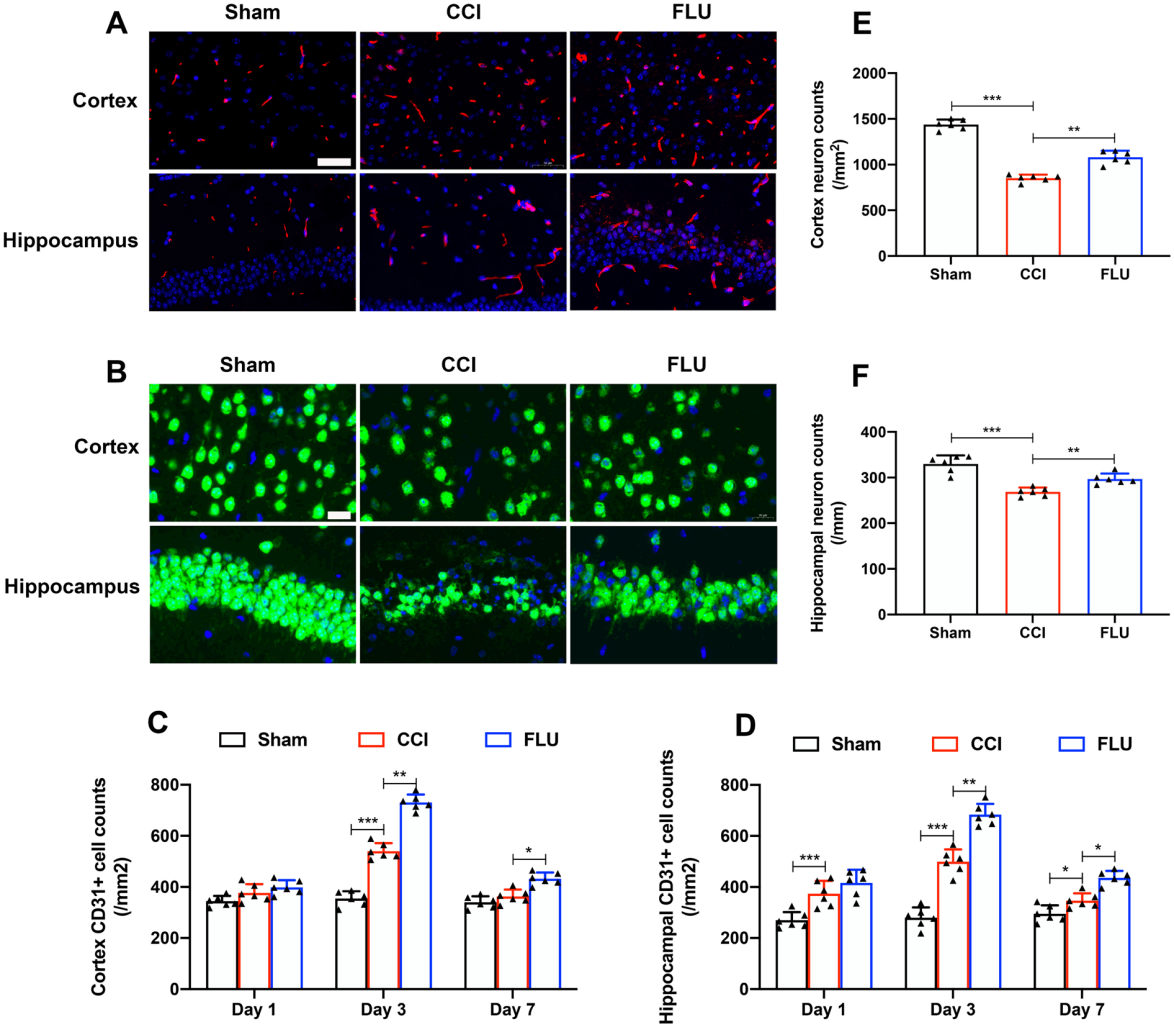
FLU treatment promoted angiogenesis and neuronal survival in the injured cortex and ipsilateral hippocampus after TBI. (A) Representative immunofluorescence images of CD31‐positive cells (Red fluorescence, scale bar: 50 μm); (B) Representative immunofluorescence images of NEUN‐positive cells (Green fluorescence, scale bar: 20 μm); (C, D) Quantification of the of CD31‐ positive cells in the injured cortex and ipsilateral hippocampus on postinjury day 1, 3 and 7; (E, F) Quantification of the of NEUN‐ positive cells in the injured cortex and ipsilateral hippocampus on postinjury day 7;. *p < 0.05, **p < 0.01, ***p < 0.001. The data are presented as the means ± SD (*n* = 6). The data are presented as the means ± SD (*n* = 6).

To evaluate the impact of FLU on angiogenesis and neuronal survival post‐TBI, we quantified VEGF expression and the counts of CD31‐positive endothelial cells on post‐injury days 1, 3, and 7, as well as NEUN‐positive cells at post‐injury day 7 in the ipsilateral cortex and hippocampus (Figure 2). Our results showed a significant increase in CD31‐positive cells around the cortical lesion site and in the ipsilateral hippocampus following TBI, with a peak on day 3 post‐injury (*p* < 0.05). FLU treatment significantly increased the number of CD31‐positive cells (*p* < 0.05) (Figure 2C,D). Concurrently, the number of NEUN‐positive cells was significantly reduced after TBI, and FLU treatment significantly increased the number of NEUN‐positive cells (*p* < 0.05) (Figure 2E,F). The data suggested that FLU promoted angiogenesis and neuronal survival after TBI.

### 3.3 FLU Increased VEGF Expression but Did Not Aggravate Brain Edema After TBI

**FIGURE 3:**
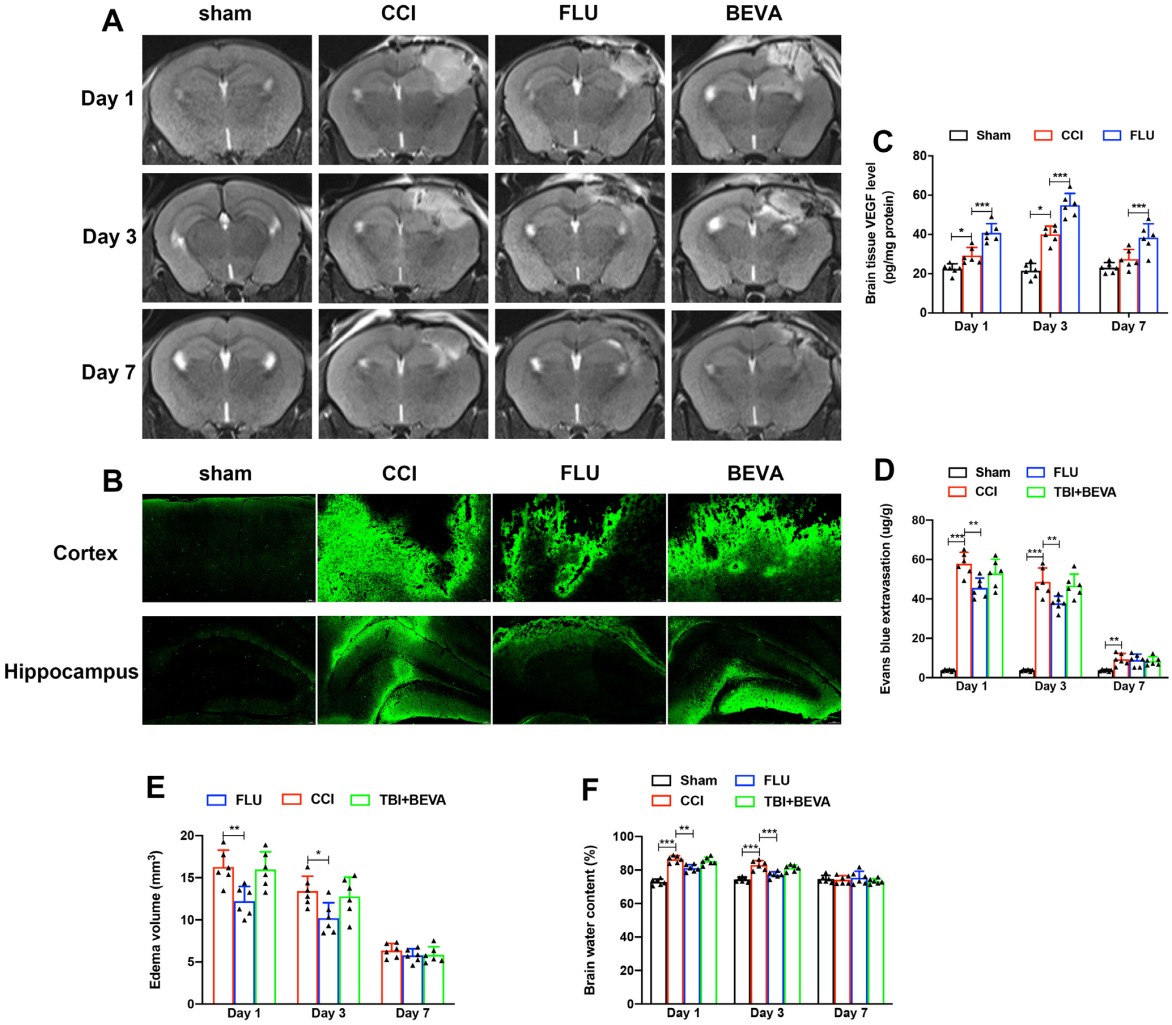
FLU increased VEGF expression but did not aggravate brain edema after TBI. (A) Representative images of T2‐weighted MRI; (B) Representative immunofluorescence images of Evans blue in the injured cortex and ipsilateral hippocampus (Green fluorescence); (C) Quantification of brain tissue VEGF level in the ipsilateral hemisphere on postinjury day 1, 3 and 7; (D) Quantification of Evan blue extravasation in the ipsilateral hemisphere on postinjury day 1, 3 and 7; (E) Quantification of edema volume around the injury site on postinjury day 1, 3 and 7; (F) Quantification of brain water content of the ipsilateral hemisphere on postinjury day 1, 3 and 7. *p < 0.05, **p < 0.01, ***p < 0.001. The data are presented as the means ± SD (*n* = 6).

Studies have suggested that VEGF can increase blood–brain barrier (BBB) permeability and potentially aggravate brain edema. To explore the effects of VEGF on BBB and brain edema, we assessed brain tissue VEGF expression, brain water content, edema volume, and EB extravasation after TBI (Figure 3). Our results showed a modest increase in VEGF expression in the ipsilateral hemisphere on post‐injury day 1, with a peak on day 3 (*p* < 0.05) (Figure 3C). Conversely, brain water content, edema volume, and Evans Blue (EB) extravasation were significantly elevated on day 1 post‐TBI (*p* < 0.05), reaching a peak, and then gradually declined by day 3, with no significant differences observed between experimental groups by day 7 (*p* > 0.05) (Figure 3D–F). Treatment with FLU significantly decreased brain water content and edema volume and concurrently increased VEGF expression (*p* < 0.05). In contrast, the VEGF antagonist BEVA did not significantly alleviate brain edema, edema volume, or EB extravasation. These data suggested that elevated VEGF expression did not exacerbate brain edema post‐TBI, FLU not only promoted angiogenesis by upregulating the VEGF expression but also reduced the brain edema.

**FIGURE 4:**
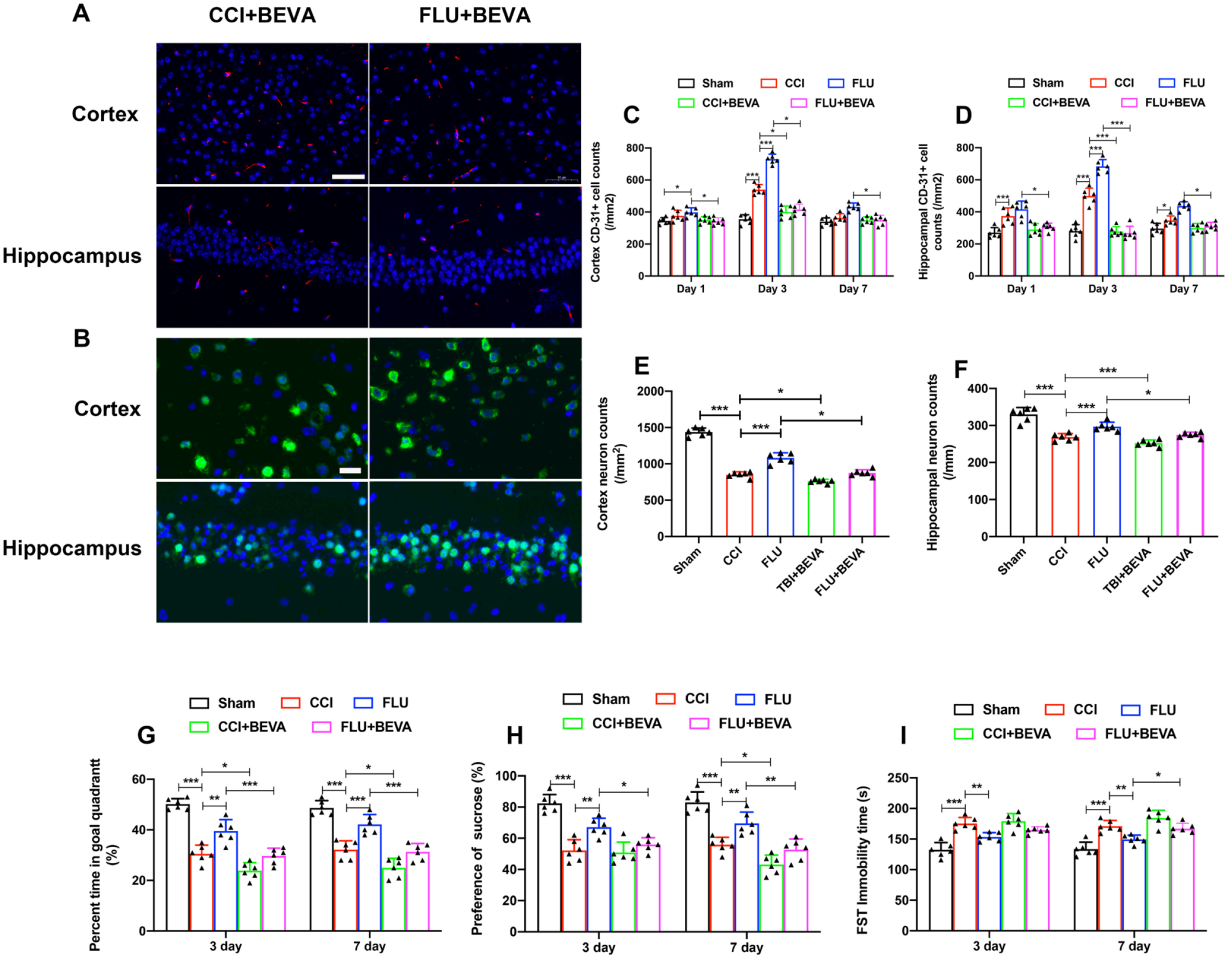
BEVA counteracted the neuroprotective effects of FLU after TBI. (A) Representative immunofluorescence images of CD31‐positive cells (Red fluorescence, scale bar: 50 μm); (B) Representative immunofluorescence images of NEUN‐positive cells (Green fluorescence, scale bar: 20 μm); (C–F) Quantification of the of CD31 and NEUN‐positive cells in the injured cortex and ipsilateral hippocampus; (G) Quantification of the percentage of time in goal quadrant on postinjury day 3 and 7; (H) Quantification of the percentage of sucrose on postinjury day 3 and 7; (I) Quantification of the FST immobility time on postinjury day 3 and 7. *p < 0.05, **p < 0.01, ***p < 0.001. The data are presented as the means ± SD (*n* = 6).

Figure 4 showed the effects of VEGF antagonist BEVA on angiogenesis, neuronal survival, and neurological functions after TBI. The results showed that BEVA treatment inhibited angiogenesis, reduced the number of neurons, and exacerbated neurological dysfunctions (*p* < 0.05). Moreover, BEVA significantly attenuated the therapeutic effects of FLU (*p* < 0.05), suggesting that FLU exerted neuroprotective effects partially by promoting angiogenesis.

### 3.4 The Role of Microglia in the FLU‐Induced Angiogenesis and Neuroprotective Effects After TBI

**FIGURE 5:**
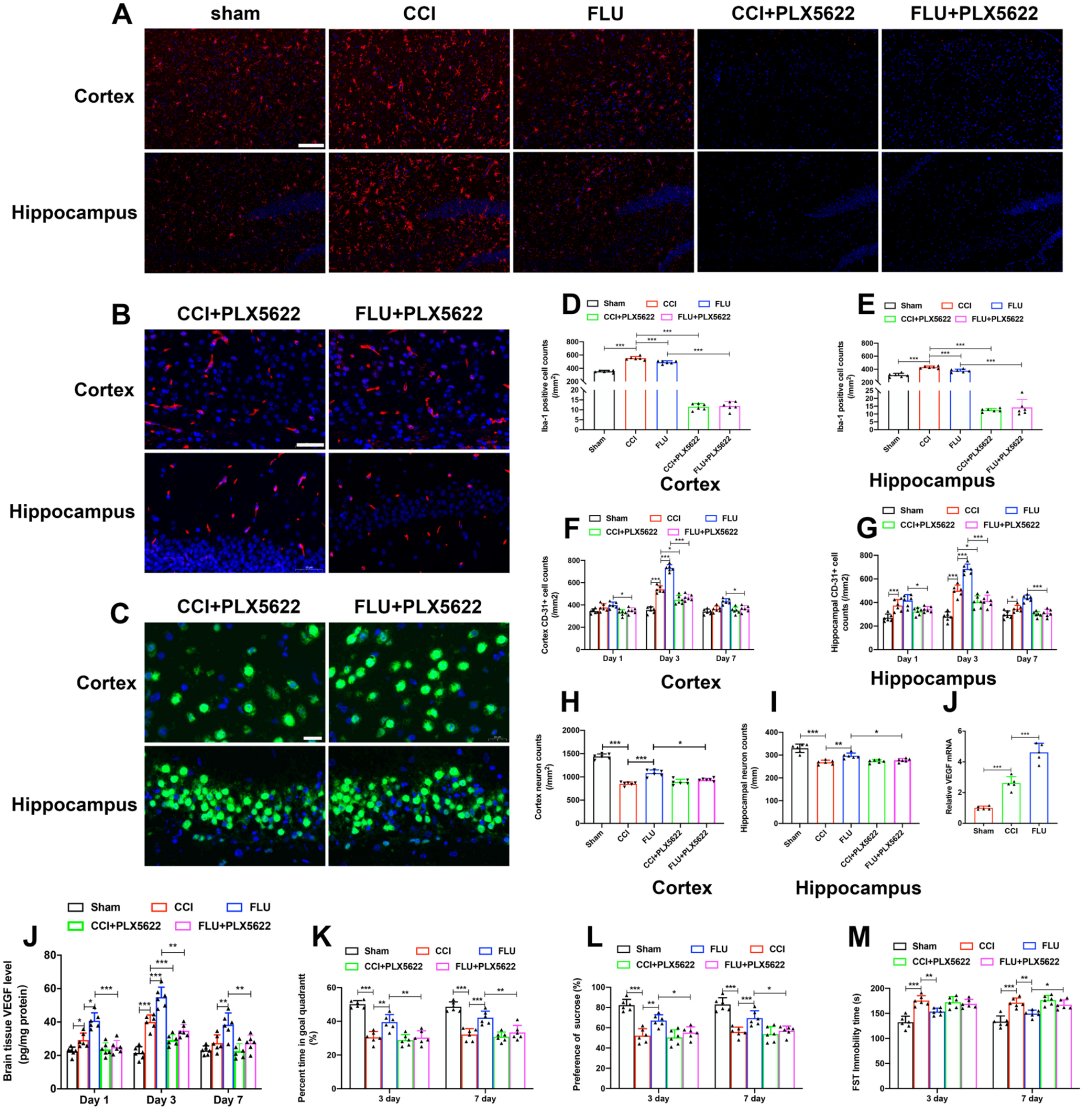
Depletion of microglia reduced FLU‐induced angiogenesis and neuroprotective effects after TBI. (A) Representative immunofluorescence images of iba‐1 positive cells (Red fluorescence, scale bar: 100 μm); (B) Representative immunofluorescence images of CD31‐positive cells (Red fluorescence, scale bar: 50 μm); (C) Representative immunofluorescence images of NEUN‐positive cells (Green fluorescence, scale bar: 20 μm); (D–I) Quantification of the Iba‐1, CD31 and NEUN‐ positive cells in the injured cortex and ipsilateral hippocampus; (J) Quantification of brain tissue VEGF level in the ipsilateral hemisphere after microglia depletion; (K) Quantification of the percentage of time in goal quadrant on postinjury day 3 and 7; (L) Quantification of the percentage of sucrose on postinjury day 3 and 7; (M) Quantification of the FST immobility time on postinjury day 3 and 7. *p < 0.05, **p < 0.01, ***p < 0.001. The data are presented as the means ± SD (*n* = 6).

Microglia have been reported to play important roles in promoting brain angiogenesis after brain injury [9, 11]. To evaluate the impact of microglia on angiogenesis, neuronal survival, and neurological functions after TBI, a continuous administration of PLX5622 was used to induce a widespread loss of microglia throughout the experimental period after TBI. As shown in Figure 5, PLX5622 treatment dramatically reduced the number of iba‐1+ cell in the cortex and hippocampus (*p* < 0.05) (Figure 5D,E). After eliminating microglia, the VEGF level and angiogenesis were significantly decreased after TBI compared to mice without microglia depletion. Furthermore, FLU treatment did not significantly alter the VEGF level and angiogenesis with absence of microglia in PLX5622‐treated mice after TBI. Accordingly, depletion of microglial did not promote neuronal survival and improve the neurological functions after TBI; instead, it weakened the angiogenesis and neuronal survival induced by FLU (Figure 5F–M). Moreover, PCR assays on isolated CD11b‐positive cells revealed that VEGF mRNA levels were significantly elevated in microglia after TBI, and FLU treatment further increased VEGF mRNA levels in microglia compared to the CCI group. Collectively, our findings suggested that microglia contributed to angiogenesis induced by FLU after TBI.

### 3.5 The Protective Effects of FLU Required the Phenotypic Transformation of Microglia After TBI

**FIGURE 6:**
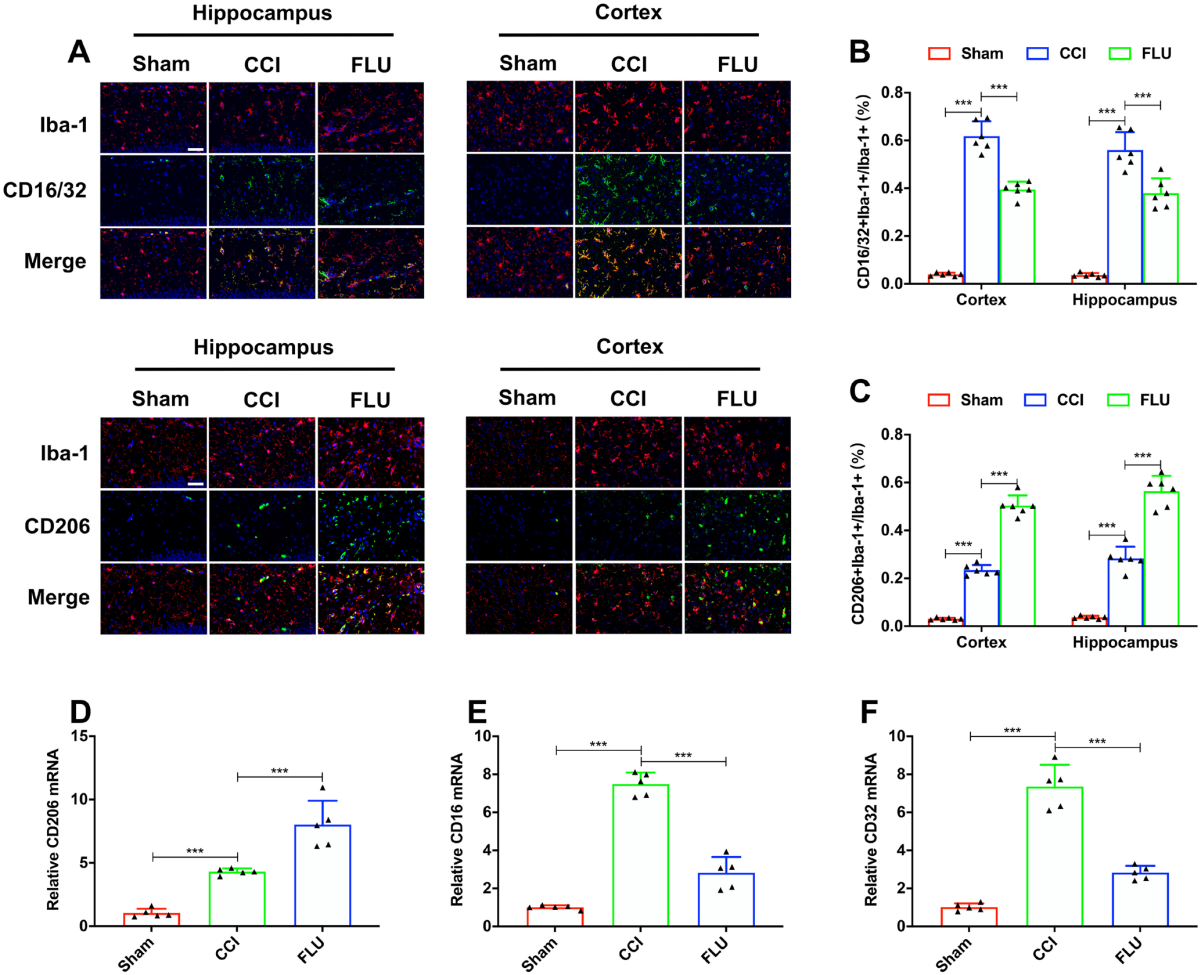
FLU treatment promoted the transformation of microglia from M1 to M2 phenotype after TBI. (A) Representative immunofluorescence images of iba‐1 (Red fluorescence), CD16/32 (Green fluorescence) and CD206 (Green fluorescence)‐positive cells in the injured cortex and ipsilateral hippocampus (Scale bar: 50 μm); (B, C) Quantification of the ratios of CD16/32 + iba‐1+/iba‐1+ and CD206 + iba‐1+/iba‐1+; (D–F) Quantification of the relative CD206, CD16 and CD32 mRNAs. *p < 0.05, **p < 0.01, ***p < 0.001. The data are presented as the means ± SD (*n* = 6).

To investigate the effects of FLU on microglial polarization and neuroinflammation following TBI, CD16/32 (M1) or CD206 (M2) was co‐labeled with the Iba‐1 (the marker of microglia/macrophage) and was counted in the ipsilateral cortex and hippocampus on post‐injury day 3. Our results showed a significant increase in the ratio of both CD16/32 + Iba‐1 double‐positive and CD206 + Iba‐1 double‐positive microglia on post‐injury day 3. FLU treatment significantly increased the number of CD206 + Iba‐1 double‐positive microglia and decreased the number of CD16/32 + Iba‐1 double‐positive microglia, indicating a shift towards the M2 phenotype (*p* < 0.05, Figure 6B,C).

**FIGURE 7:**
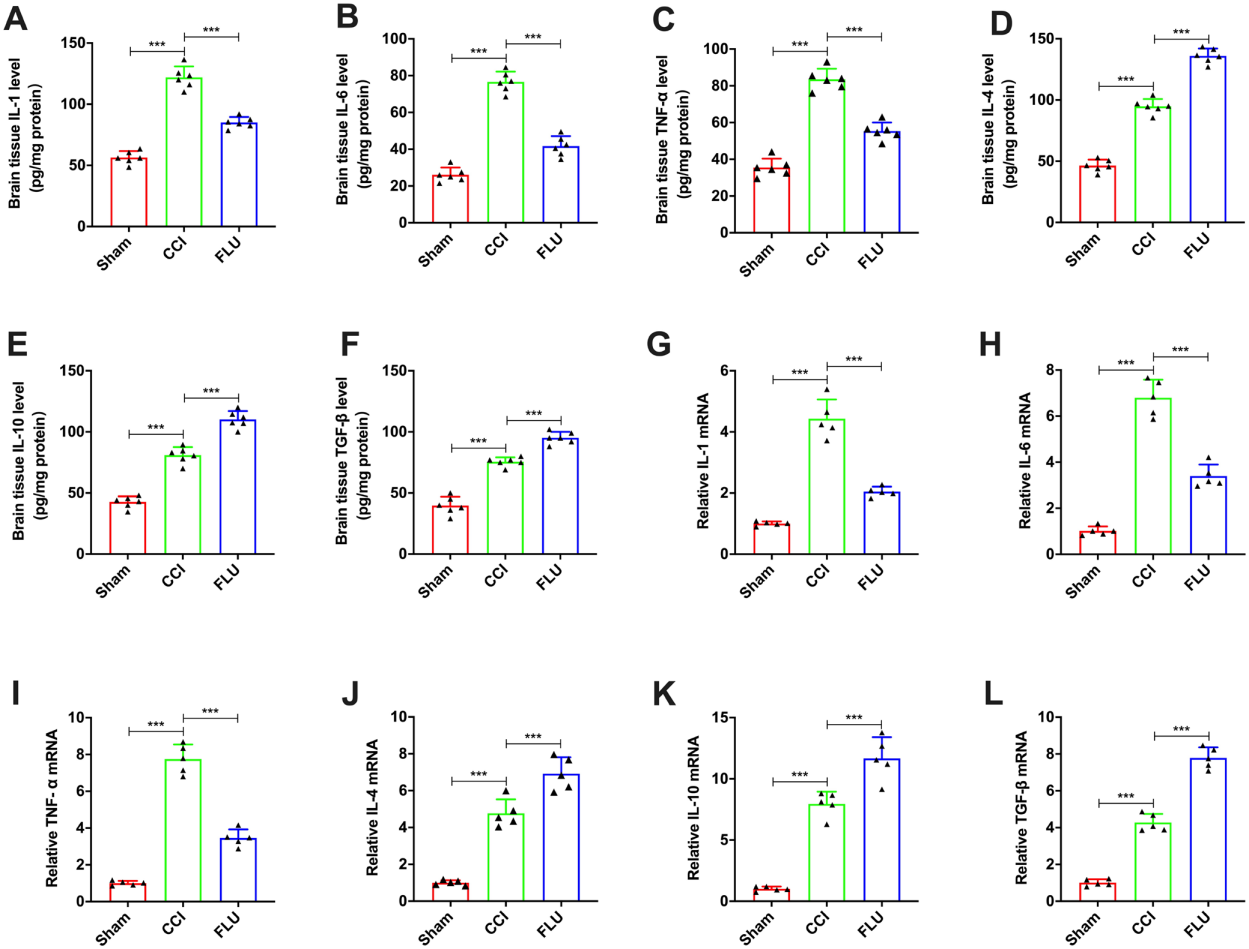
Effects of FLU on the expression of inflammatory cytokines in brain tissue and isolated microglia in the ipsilateral hemisphere after TBI. (A–F) Quantification of brain tissue IL‐1, IL‐6, TNF‐α, IL‐4, IL‐10 and TGF‐β in the ipsilateral hemisphere; (G‐L) Quantification of the relative IL‐1, IL‐6, TNF‐α, IL‐4, IL‐10 and TGF‐β mRNAs in the isolated microglia. *p < 0.05, **p < 0.01, ***p < 0.001. The data are presented as the means ± SD (*n* = 6 for Elisa and *n* = 5 for PCR).

In addition, we also evaluated the levels of the signature proteins of pro‐inflammatory (TNF‐α, IL‐1β and IL‐6) and anti‐inflammatory (IL‐14, IL‐10, TGF‐β) in the ipsilateral hemisphere. ELISA results showed that both pro‐inflammatory and anti‐inflammatory signature proteins were remarkably increased at 3 days after TBI. Compared with the CCI group, FLU treatment caused a significant decrease in pro‐inflammatory signature proteins, while increased the expression of anti‐inflammatory signature proteins (*p* < 0.05, Figure 7A–F).

Accordingly, PCR assay of CD11b‐positive cells isolated from the injury site indicated that FLU treatment increased mRNA levels of M2‐associated markers, including CD206, IL‐4, IL‐10, and TGF‐β1, whereas it reduced the M1‐associated markers, such as CD16, CD32, and TNF‐α (*p* < 0.05, Figure 7G–L). Taken together, these results suggested that FLU treatment promoted the pro‐inflammatory to anti‐inflammatory phenotypic shift of microglia, which is involved in the process of angiogenesis after TBI.

### 3.6 The Signal Pathways Involved in FLU‐Induced M2 Polarization After TBI

**FIGURE 8:**
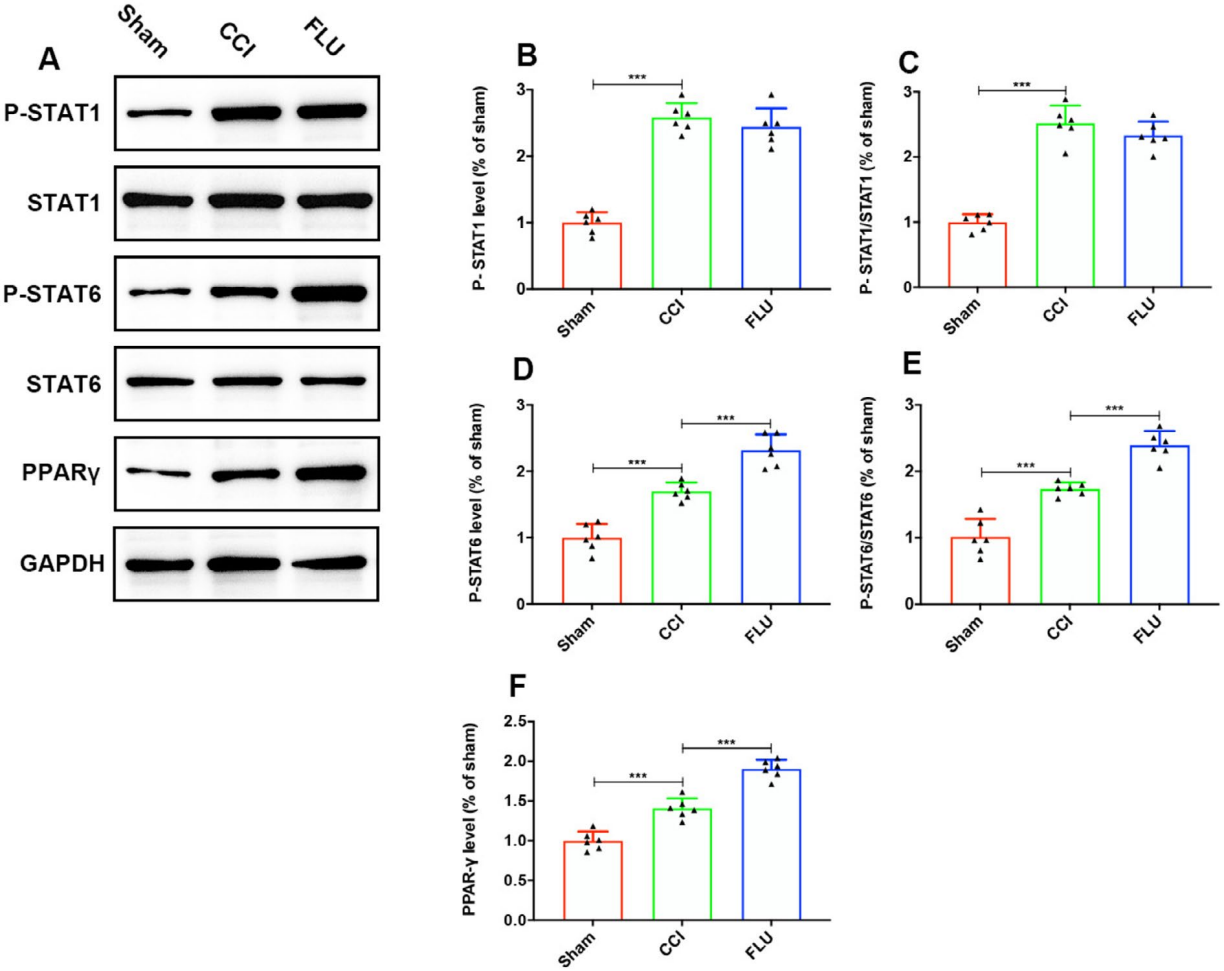
FLU regulated the phenotypic shift of microglia from M1 to M2 via JAK/STAT6/PPARγ pathway after TBI. (A) Representative western blot images showing P‐STAT1, STAT1, P‐STAT6, STAT6, and PPARγ expression; (B–F) Quantification of P‐STAT1, P‐STAT1/STAT1, P‐STAT6, P‐STAT6/STAT6 and PPARγ. The relative band density was measured with ImageJ (1.49 V) and normalized to that of GAPDH, and the percent expression compared to that of sham controls was calculated for each sample. *p < 0.05, **p < 0.01, ***p < 0.001. The data are presented as the means ± SD (*n* = 6).

JAK/Signal Transducer and Activator of Transcription (STAT) pathways have been reported to play important roles in the phenotypic switching of microglia and macrophages. Specifically, STAT1 has been shown to promote M1 polarization, while STAT6 and STAT3 promote M2 polarization in response to IL‐4 and IL‐10 stimulation, respectively. Additionally, agonists of the Peroxisome Proliferator‐Activated Receptor Gamma (PPARγ) have been demonstrated to induce a transition of microglia from the M1 to M2 phenotype. To clarify the signaling pathways involved in FLU‐mediated microglia phenotypic switching, we examined the effects of FLU on the activity of JAK/STAT pathways after TBI. Our results showed that P‐STAT1, P‐STAT1/STAT1, P‐STAT6, P‐STAT6/STAT6 and the expression level of PPARγ in the ipsilateral hemisphere were significantly increased on post‐injury day 3. FLU treatment significantly increased P‐STAT6/STAT6 but not P‐STAT1/STAT1, and increased the expression of PPARγ (*p* < 0.05, Figure 8). Figure S1 showed the original gel/blot images. These results suggested that FLU may facilitate the transition of microglia towards an M2 phenotype by modulating the JAK/STAT 6/PPARγ pathway expression.

### 3.7 The Effects of MR on Angiogenesis Are Mediated via PI3K/AKT/HIF‐1α/VEGF Signal Pathway

**FIGURE 9:**
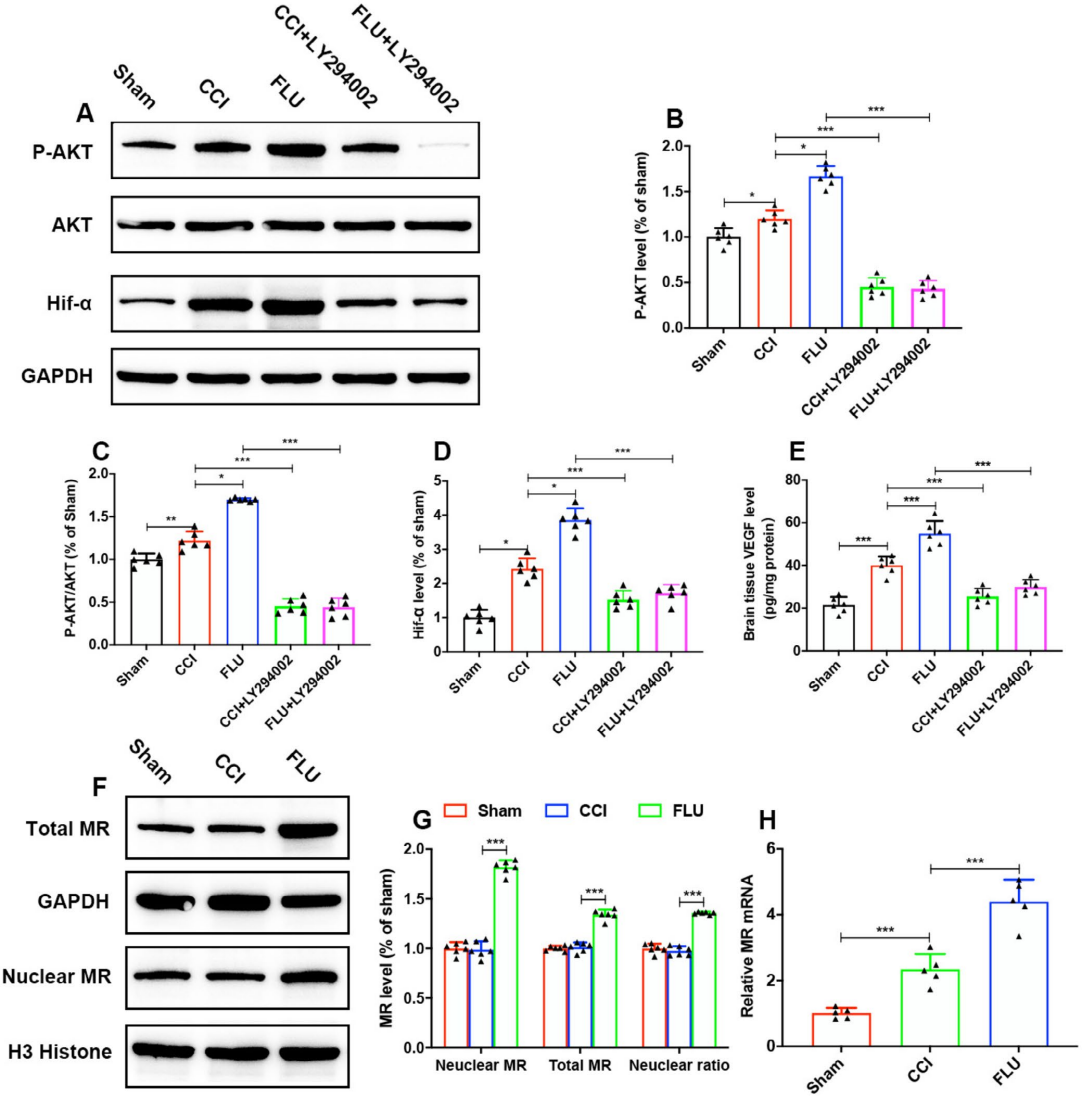
FLU increased angiogenesis by activating MR via PI3K/Akt/Hif‐1α signaling pathway after TBI. (A) Representative western blot images showing p‐Akt, Akt, and Hif‐1α expression; (B–E) Quantification of p‐Akt, p‐Akt/Akt, Hif‐1α and VEGF levels; (F) Representative western blot images showing nuclear and total MR; (G) Quantification of nuclear MR, total MR, and the ratio of nuclear/total MR; (H) Quantification of the relative MR mRNAs in the isolated microglia. The relative band density was measured with ImageJ (1.49 V) and normalized to that of GAPDH, and the percent expression compared to that of sham controls was calculated for each sample. *p < 0.05, **p < 0.01, ***p < 0.001. The data are presented as the means ± SD (*n* = 6).

The PI3K/AKT/HIF‐1α signaling pathway is crucial in the regulation of VEGF expression and plays important roles in angiogenesis under both normoxic and hypoxic conditions. To investigate the activity of this pathway and its influence on VEGF expression following TBI and FLU treatment, we assessed the levels of phosphorylated and total AKT (P‐Akt/Akt), hypoxia‐inducible factor‐1 alpha (HIF‐1α) and VEGF expressions in the ipsilateral hemisphere on post‐injury day 3. The results showed that FLU treatment significantly increased the P‐Akt, P‐Akt/Akt, HIF‐1 and VEGF expressions by activating MR (*p* < 0.05, Figure 9). Conversely, treatment with the PI3K inhibitor LY294002 counteracted the effects of FLU, leading to a significant reduction in the ratios of P‐Akt/Akt and the expression levels of HIF‐1α and VEGF (*p* < 0.05), suggesting that FLU regulated VEGF expression and angiogenesis via PI3K/AKT/HIF‐1α pathway. Figure S1 showed the original gel/blot images.

## 4 Discussion

In the present study, we investigated the effects of MR activation by FLU on microglial polarization, angiogenesis, neuronal survival, functional rehabilitation, and the possible signaling pathways in mice after TBI. We found that the MR specific agonist FLU promoted the transformation of microglia from the pro‐inflammatory M1 phenotype to the anti‐inflammatory M2 phenotype through the JAK/STAT6/PPARγ pathway. FLU treatment effectively inhibited neuroinflammatory responses, increased VEGF expression, stimulated angiogenesis, promoted neuronal survival, and improved functional rehabilitation by upregulating the expression and activation level of MR after TBI. Moreover, we identified the PI3K/Akt/HIF‐1α pathway as a key player in VEGF expression and the subsequent angiogenesis. Our results suggested that FLU did not only exert its anti‐inflammatory effects but also promoted neurorepair by modification of the balance between M1 and M2 phenotypes of microglia rather than a complete suppression of microglial activity.

Following TBI, both peripheral and innate immune cells participate in the acute neuroinflammatory response around the injury site [1, 3]. Microglia, as the primary immune cells resident in the brain, serve as the first line of defense against injuries. Upon the initial mechanical damage after TBI, microglia immediately initiate a cascade of immune events triggered by the release of DAMPs [31, 32, 33, 34], which cause the recruitment of peripheral phagocytes, such as monocyte‐derived macrophages and neutrophils, into the brain from the blood. Neutrophils, the first peripheral immune cells to arrive at the injury site, peak within 24 h and then decline dramatically within several days. Studies have shown that neutrophil accumulation promoted the secondary injury cascades and exerted an overall neurotoxic effect [35]. In contrast, microglia are mobilized within hours and continue to accumulate for months or even years. Besides promoting inflammation, they are among the most potent modulators of CNS repair and regeneration. An experimental study revealed a 10‐ to 20‐fold increase in microglia numbers compared to the peripheral macrophages after focal brain injury, indicating a central response rather than a peripheral one around the injury site after TBI [36]. Accumulating evidence suggests that activated microglia hinder or promote neurological recovery, depending on their phenotypes under different microenvironments, such as proinflammatory (M1) and anti‐inflammatory (M2) phenotypes [6, 7, 8, 9, 10, 11]. The M1 phenotype, marked by signature proteins like CD16 and CD32, tends to release destructive mediators; the M2 phenotype, characterized by CD206 and arginase 1, releases neurotrophic factors such as VEGF and BDNF, which support brain tissue repair, including angiogenesis and neurogenesis. In this regard, identification of the molecular switches and drugs that trigger phenotypic shifts could offer promising clinical therapeutic strategies for brain injury. Accordingly, our results showed that microglia were activated after TBI; both the number of M1 and M2 microglia increased around the lesion. The activated microglia promoted neuroinflammation by transforming into the M1 phenotype and upregulating the pro‐inflammatory factors on one hand, and on the other hand, they also exerted anti‐inflammatory effects and promoted neurorepair by transforming into the M2 phenotype, which released the anti‐inflammatory factors and neurotrophic factors. To test the hypothesis that suppressing all microglia would not be beneficial after TBI, we depleted most of the microglia by PLX5622 before TBI and observed no improvement in neuronal survival and neurofunction after TBI. In contrast, microglia depletion weakened the FLU‐induced angiogenesis and neuroprotective effects, suggesting that microglia were involved in the FLU‐induced neuroprotection after TBI.

GR agonists like DEX and MP have been extensively utilized in TBI patients to suppress neuroinflammation, alleviate vasogenic edema, and reduce elevated intracranial pressure [37, 38, 39]. However, subsequent studies have not demonstrated an overall benefit from high‐dose GCs, with the mechanisms underlying their effects remaining elusive [40, 41]. We previously revealed that DEX exerted potent anti‐inflammatory effects by inhibiting the activation of microglia and reducing the number of proinflammatory microglia; on the one hand, it also hindered angiogenesis and neurofunctional recovery by decreasing the population of anti‐inflammatory microglia after TBI [21, 22, 23, 25]. In contrast, the application of FLU, the MR agonist, has been shown to enhance neuronal survival and facilitate functional rehabilitation, although the mechanisms are not fully understood [28]. It is established that both GR and MR are highly expressed in adult microglia, making them direct targets for glucocorticoids and key regulators of microglial inflammatory responses [42, 43]. However, the impact of MR on microglial phenotypic shifts is not well characterized and warrants further investigation. Our study showed that FLU treatment not only exerted anti‐inflammatory effects by reducing M1 microglia and downregulating pro‐inflammatory signature proteins, but also played crucial roles in the neurorepair processes, including angiogenesis and neuronal survival, by increasing M2 microglia and upregulating the expression of anti‐inflammatory and neurotrophic factors. Taken together, these results suggested that FLU treatment played a dual role in mitigating neuroinflammation and promoting neurorepair by facilitating the phenotypic shift of microglia from M1 to M2 polarization.

The phenotypic shifts of microglia can be triggered by extracellular molecules from injured tissue, such as TNF, IL6, TGFβ, and IL‐4 released by injured brain tissue and peripheral immune cells after TBI [44, 45]. Multiple intracellular signaling pathways have been implicated in the phenotypic transformation of microglia in response to these factors. Notably, the STAT family of transcription factors plays a pivotal role in this process. Specifically, STAT1 increases M1 polarization after IFNγ or LPS stimulation, while STAT6 is crucial for M2 phenotype transforming after IL‐4 exposure [6, 46, 47]. Additionally, the nuclear hormone receptor PPARγ is another key modulator of microglial and macrophage phenotypes. Experimental studies have shown that PPARγ agonists provide neuroprotection by reducing inflammation and facilitating the switch from M1 to M2 microglia in CNS disease models associated with inflammation [48, 49]. Consistent with these studies, our study indicated that the phosphorylation levels of both STAT1 and STAT6 in the ipsilateral cortex and hippocampus were increased after TBI. FLU treatment significantly elevated phosphorylated STAT6 levels and enhanced PPARγ activity. The above results suggested that the JAK/STAT6/PPARγ pathway might be involved in the MR induced M1‐M2 phenotypic transformation of microglia.

Both macrovascular ruptures caused by mechanical force and microvascular injury, such as the breakdown of the BBB caused by neuroinflammation, can result in local cerebral blood flow disturbances, hypoxia‐ischemia, corresponding vasogenic and cytotoxic edema, intracranial hypertension, and brain herniation. Angiogenesis, the formation of new blood vessels, is a critical tissue repair response that is activated following brain injury [50]. Accumulating evidence has revealed that angiogenesis is essential for restoring tissue perfusion, enhancing oxygen and nutrient supply, facilitating neuronal restoration, and promoting functional rehabilitation after stroke and TBI [51, 52, 53, 54]. VEGF, a potent angiogenic factor, plays key roles in angiogenesis [55, 56]. However, some studies suggest that increased VEGF expression can exacerbate vasogenic brain edema by increasing cerebrovascular permeability after brain injury [57, 58, 59]. This implies that elevated VEGF levels could both worsen edema and promote neurorepair through enhanced angiogenesis [29]. Therefore, the overall effects of VEGF and angiogenesis after TBI require further investigation. Our study revealed that brain edema peaked at 24 h post‐TBI, while VEGF expression and angiogenesis were most pronounced at 72 h. The VEGF antagonist BEVA inhibited angiogenesis but did not reduce brain edema; instead, it decreased neuronal survival and hindered neurological recovery post‐TBI. These findings suggest that increased VEGF and angiogenesis generally play a beneficial role after TBI. Furthermore, FLU treatment unregulated VEGF expression and increased angiogenesis in the ipsilateral cortex and hippocampus after TBI. However, microglial depletion by PLX5622 reduced VEGF expression and angiogenesis induced by FLU. Notably, VEGF mRNA levels in isolated microglia were significantly increased after FLU administration, which suggested that FLU promoted angiogenesis by inducing the M1‐to‐M2 phenotype switch in microglia, positioning it as a promising therapeutic strategy for TBI.

A growing body of evidence revealed the role of HIF‐1α in facilitating neurorepair and functional recovery following stroke and TBI by upregulating VEGF expression and promoting angiogenesis [60, 61, 62]. Previous studies have shown that HIF‐1α is regulated by the PI3K/Akt signaling pathway both under normoxic conditions and hypoxic conditions; growth factors and cytokines regulate the PI3K/Akt and increase HIF‐1α levels. Moreover, the PI3K/Akt pathway is involved in VEGF‐induced angiogenesis [63, 64]. However, whether MR activation upregulates VEGF expression in the microglia by the PI3K/Akt/HIF‐1α pathway after TBI remains unclear. Our study showed that the levels of p‐AKT and HIF‐1α were increased surrounding the lesion after TBI, and the administration of FLU significantly increased p‐AKT and HIF‐1α, and its effects were blocked by the PI3K inhibitor LY294002. The above data suggested that the PI3K/Akt/HIF‐1α pathway played important roles in the expression of VEGF and angiogenesis after TBI, and FLU treatment regulated the expression of VEGF and angiogenesis in the microglia via the PI3K/AKT/HIF‐1α pathway.

### 4.1 Limitations of This Study

It is important to acknowledge the limitations present in this study. Firstly, although previous studies indicate that microglia account for a 10‐ to 20‐fold increase compared to peripheral macrophages after TBI, the signature proteins we used in the present study are not specific enough to differentiate between microglia and peripheral macrophages. Secondly, we used FLU to activate MR by not using knockdown and overexpression approaches. Additionally, both pro‐inflammatory M1‐like microglia and anti‐inflammatory M2‐like microglia can be characterized by the expression of some signature proteins. For example, TNF, iNOS, MHC‐II, CD16/32, and CD86 have been used to label M1‐like microglia, while arginase‐1, CD206, and TGF‐β have been used to label M2‐like microglia. Our study only used iba‐1 + CD16/32 double‐positive cells to define M1‐like microglia, and iba‐1 + CD206 positive cells to define M2‐like microglia. Moreover, we only used male rats to exclude the effects of sex and sex hormones on glucocorticoids and their receptors. In our following study, we will further investigate the effects of sex and sex hormones on MR‐induced neuroprotective effects.

## 5 Conclusions

In conclusion, our study indicated that MR was abundantly expressed in microglia and played a pivotal role in modulating their inflammatory responses after TBI. FLU, the specific MR agonist, exerts anti‐neuroinflammatory and neuroprotective effects by regulating phenotypic switching of microglia. Instead of suppressing all types of microglia, FLU treatment induced the transformation of M1‐like microglia to M2‐like microglia via the JAK/STAT6/PPARγ pathway, promoted VEGF expression and angiogenesis via the PI3K/Akt/HIF‐1α pathway, increased the number of survival neurons, and improved functional rehabilitation after TBI. Our study provided a theoretical basis for a potential therapeutic strategy targeting neuroinflammation and neurorepair after TBI.

## Conflicts of Interest

The authors declare no conflicts of interest.

## Supporting information


Figure S1.

## Data Availability

The data that support the findings of this study are available from the corresponding author upon reasonable request.

## References

[1] M. C. Morganti‐Kossmann , B. D. Semple , S. C. Hellewell , N. Bye , and J. M. Ziebell , “The Complexity of Neuroinflammation Consequent to Traumatic Brain Injury: From Research Evidence to Potential Treatments,” Acta Neuropathologica 137, no. 5 (2019): 731–755. 30535946 10.1007/s00401-018-1944-6

[2] D. W. Simon , M. J. McGeachy , H. Bayır , R. S. Clark , D. J. Loane , and P. M. Kochanek , “The Far‐Reaching Scope of Neuroinflammation After Traumatic Brain Injury,” Nature Reviews Neurology 13, no. 3 (2017): 171–191. 28186177 10.1038/nrneurol.2017.13 PMC5675525

[3] A. Alam , E. P. Thelin , T. Tajsic , et al., “Cellular Infiltration in Traumatic Brain Injury,” Journal of Neuroinflammation 17, no. 1 (2020): 328. 33143727 10.1186/s12974-020-02005-x PMC7640704

[4] Y. N. Jassam , S. Izzy , M. Whalen , D. B. McGavern , and J. El Khoury , “Neuroimmunology of Traumatic Brain Injury: Time for a Paradigm Shift,” Neuron 95, no. 6 (2017): 1246–1265. 28910616 10.1016/j.neuron.2017.07.010 PMC5678753

[5] K. Borst , A. A. Dumas , and M. Prinz , “Microglia: Immune and non‐immune functions,” Immunity 54, no. 10 (2021): 2194–2208. 34644556 10.1016/j.immuni.2021.09.014

[6] H. Wu , J. Zheng , S. Xu , et al., “Mer Regulates Microglial/Macrophage M1/M2 Polarization and Alleviates Neuroinflammation Following Traumatic Brain Injury,” Journal of Neuroinflammation 18, no. 1 (2021): 2. 33402181 10.1186/s12974-020-02041-7 PMC7787000

[7] Z. Li , J. Xiao , X. Xu , et al., “M‐CSF, IL‐6, and TGF‐β Promote Generation of a New Subset of Tissue Repair Macrophage for Traumatic Brain Injury Recovery,” Science Advances 7, no. 11 (2021): eabb6260. 33712456 10.1126/sciadv.abb6260 PMC7954455

[8] S. R. Var , A. V. Shetty , A. W. Grande , W. C. Low , and M. C. Cheeran , “Microglia and Macrophages in Neuroprotection, Neurogenesis, and Emerging Therapies for Stroke,” Cells 10, no. 12 (2021): 3555. 34944064 10.3390/cells10123555 PMC8700390

[9] S. Li , X. Hua , M. Zheng , et al., “PLXNA2 Knockdown Promotes M2 Microglia Polarization Through mTOR/STAT3 Signaling to Improve Functional Recovery in Rats After Cerebral Ischemia/Reperfusion Injury,” Experimental Neurology 346 (2021): 113854. 34474008 10.1016/j.expneurol.2021.113854

[10] Y. F. Li , X. Ren , L. Zhang , Y. H. Wang , and T. Chen , “Microglial Polarization in TBI: Signaling Pathways and Influencing Pharmaceuticals,” Frontiers in Aging Neuroscience 14 (2022): 901117. 35978950 10.3389/fnagi.2022.901117 PMC9376354

[11] X. Hu , R. K. Leak , Y. Shi , et al., “Microglial and Macrophage Polarization—New Prospects for Brain Repair,” Nature Reviews. Neurology 11, no. 1 (2015): 56–64. 25385337 10.1038/nrneurol.2014.207 PMC4395497

[12] A. Jacquens , E. J. Needham , E. R. Zanier , V. Degos , P. Gressens , and D. Menon , “Neuro‐Inflammation Modulation and Post‐Traumatic Brain Injury Lesions: From Bench to Bed‐Side,” International Journal of Molecular Sciences 23, no. 19 (2022): 11193. 36232495 10.3390/ijms231911193 PMC9570205

[13] S. Kalra , R. Malik , G. Singh , et al., “Pathogenesis and Management of Traumatic Brain Injury (TBI): Role of Neuroinflammation and Anti‐Inflammatory Drugs,” Inflammopharmacology 30, no. 4 (2022): 1153–1166. 35802283 10.1007/s10787-022-01017-8 PMC9293826

[14] J. Renaudin , D. Fewer , C. B. Wilson , E. B. Boldrey , J. Calogero , and K. J. Enot , “Dose Dependency of Decadron in Patients With Partially Excised Brain Tumors,” Journal of Neurosurgery 39, no. 3 (1973): 302–305. 4733430 10.3171/jns.1973.39.3.0302

[15] H. M. Reichardt , R. Gold , and F. Lühder , “Glucocorticoids in multiple sclerosis and experimental autoimmune encephalomyelitis,” Expert Review of Neurotherapeutics 6, no. 11 (2006): 1657–1670. 17144780 10.1586/14737175.6.11.1657

[16] M. B. Bracken , M. J. Shepard , W. F. Collins , et al., “A Randomized, Controlled Trial of Methylprednisolone or Naloxone in the Treatment of Acute Spinal‐Cord Injury. Results of the Second National Acute Spinal Cord Injury Study,” New England Journal of Medicine 322, no. 20 (1990): 1405–1411. 2278545 10.1056/NEJM199005173222001

[17] P. Edwards , M. Arango , L. Balica , et al., “Final Results of MRC CRASH, a Randomised Placebo‐Controlled Trial of Intravenous Corticosteroid in Adults With Head Injury‐Outcomes at 6 Months,” Lancet 365, no. 9475 (2005): 1957–1959. 15936423 10.1016/S0140-6736(05)66552-X

[18] E. R. de Kloet , O. C. Meijer , A. F. de Nicola , R. H. de Rijk , and M. Joëls , “Importance of the Brain Corticosteroid Receptor Balance in Metaplasticity, Cognitive Performance and Neuro‐Inflammation,” Frontiers in Neuroendocrinology 49 (2018): 124–145. 29428549 10.1016/j.yfrne.2018.02.003

[19] A. P. Harris , M. C. Holmes , E. R. de Kloet , K. E. Chapman , and J. R. Seckl , “Mineralocorticoid and Glucocorticoid Receptor Balance in Control of HPA Axis and Behaviour,” Psychoneuroendocrinology 38, no. 5 (2013): 648–658. 22980941 10.1016/j.psyneuen.2012.08.007

[20] E. R. de Kloet , “Brain Mineralocorticoid and Glucocorticoid Receptor Balance in Neuroendocrine Regulation and Stress‐Related Psychiatric Etiopathologies,” Current Opinion in Endocrine and Metabolic Research 24 (2022): 100352. 38037568 10.1016/j.coemr.2022.100352 PMC10687720

[21] B. Zhang , X. Xu , F. Niu , et al., “Corticosterone Replacement Alleviates Hippocampal Neuronal Apoptosis and Spatial Memory Impairment Induced by Dexamethasone via Promoting Brain Corticosteroid Receptor Rebalance After Traumatic Brain Injury,” Journal of Neurotrauma 37, no. 2 (2020): 262–272. 31436134 10.1089/neu.2019.6556

[22] B. Zhang , M. Bai , X. Xu , et al., “Corticosteroid Receptor Rebalancing Alleviates Critical Illness‐Related Corticosteroid Insufficiency After Traumatic Brain Injury by Promoting Paraventricular Nuclear Cell Survival via Akt/CREB/BDNF Signaling,” Journal of Neuroinflammation 17, no. 1 (2020): 318. 33100225 10.1186/s12974-020-02000-2 PMC7586672

[23] B. Zhang , X. Zhu , L. Wang , et al., “Dexamethasone Impairs Neurofunctional Recovery in Rats Following Traumatic Brain Injury by Reducing Circulating Endothelial Progenitor Cells and Angiogenesis,” Brain Research 1725 (2019): 146469. 31541641 10.1016/j.brainres.2019.146469

[24] A. Sierra , A. Gottfried‐Blackmore , T. A. Milner , B. S. McEwen , and K. Bulloch , “Steroid Hormone Receptor Expression and Function in Microglia,” Glia 56, no. 6 (2008): 659–674. 18286612 10.1002/glia.20644

[25] M. Yang , M. Bai , Y. Zhuang , et al., “High‐Dose Dexamethasone Regulates Microglial Polarization via the GR/JAK1/STAT3 Signaling Pathway After Traumatic Brain Injury,” Neural Regeneration Research 20, no. 9 (2025): 2611–2623. 39314167 10.4103/NRR.NRR-D-23-01772 PMC11801282

[26] H. Yang , S. Narayan , and M. V. Schmidt , “From Ligands to Behavioral Outcomes: Understanding the Role of Mineralocorticoid Receptors in Brain Function,” Stress 26, no. 1 (2023): 2204366. 37067948 10.1080/10253890.2023.2204366

[27] M. E. Brocca , L. Pietranera , E. R. de Kloet , and A. F. De Nicola , “Mineralocorticoid Receptors, Neuroinflammation and Hypertensive Encephalopathy,” Cellular and Molecular Neurobiology 39, no. 4 (2019): 483–492. 30117098 10.1007/s10571-018-0610-9 PMC11469880

[28] B. Zhang , X. Zhu , L. Wang , et al., “Inadequate Expression and Activation of Mineralocorticoid Receptor Aggravates Spatial Memory Impairment After Traumatic Brain Injury,” Neuroscience 424 (2020): 1–11. 31734415 10.1016/j.neuroscience.2019.10.026

[29] M. Tado , T. Mori , M. Fukushima , et al., “Increased Expression of Vascular Endothelial Growth Factor Attenuates Contusion Necrosis Without Influencing Contusion Edema After Traumatic Brain Injury in Rats,” Journal of Neurotrauma 31, no. 7 (2014): 691–698. 24294928 10.1089/neu.2013.2940

[30] W. Yao , Q. Cao , S. Luo , et al., “Microglial ERK‐NRBP1‐CREB‐BDNF Signaling in Sustained Antidepressant Actions of (R)‐ketamine,” Molecular Psychiatry 27, no. 3 (2022): 1618–1629. 34819637 10.1038/s41380-021-01377-7 PMC9095473

[31] E. F. Willis , K. P. A. MacDonald , Q. H. Nguyen , et al., “Repopulating Microglia Promote Brain Repair in an IL‐6‐Dependent Manner,” Cell 180, no. 5 (2020): 833–846. 32142677 10.1016/j.cell.2020.02.013

[32] K. G. Witcher , C. E. Bray , T. Chunchai , et al., “Traumatic Brain Injury Causes Chronic Cortical Inflammation and Neuronal Dysfunction Mediated by Microglia,” Journal of Neuroscience 41, no. 7 (2021): 1597–1616. 33452227 10.1523/JNEUROSCI.2469-20.2020 PMC7896020

[33] D. Davalos , J. Grutzendler , G. Yang , et al., “ATP Mediates Rapid Microglial Response to Local Brain Injury In Vivo,” Nature Neuroscience 8, no. 6 (2005): 752–758. 15895084 10.1038/nn1472

[34] A. Nimmerjahn , F. Kirchhoff , and F. Helmchen , “Resting Microglial Cells Are Highly Dynamic Surveillants of Brain Parenchyma In Vivo,” Science 308, no. 5726 (2005): 1314–1318. 15831717 10.1126/science.1110647

[35] R. S. Clark , J. K. Schiding , S. L. Kaczorowski , D. W. Marion , and P. M. Kochanek , “Neutrophil Accumulation After Traumatic Brain Injury in Rats: Comparison of Weight Drop and Controlled Cortical Impact Models,” Journal of Neurotrauma 11, no. 5 (1994): 499–506. 7861443 10.1089/neu.1994.11.499

[36] S. Gyoneva and R. M. Ransohoff , “Inflammatory Reaction After Traumatic Brain Injury: Therapeutic Potential of Targeting Cell‐Cell Communication by Chemokines,” Trends in Pharmacological Sciences 36, no. 7 (2015): 471–480. 25979813 10.1016/j.tips.2015.04.003 PMC4485943

[37] A. R. Dick , M. E. McCallum , J. A. Maxwell , and R. Nelson , “Effect of Dexamethasone on Experimental Brain Edema in Cats,” Journal of Neurosurgery 45, no. 2 (1976): 141–147, 10.3171/jns.1976.45.2.0141. 939972

[38] E. D. Hall , “The Neuroprotective Pharmacology of Methylprednisolone,” Journal of Neurosurgery 76, no. 1 (1992): 13–22. 1727150 10.3171/jns.1992.76.1.0013

[39] N. M. Dearden , J. S. Gibson , D. G. McDowall , R. M. Gibson , and M. M. Cameron , “Effect of High‐Dose Dexamethasone on Outcome From Severe Head Injury,” Journal of Neurosurgery 64, no. 1 (1986): 81–88. 3510286 10.3171/jns.1986.64.1.0081

[40] S. L. Giannotta , M. H. Weiss , M. L. Apuzzo , and E. Martin , “High Dose Glucocorticoids in the Management of Severe Head Injury,” Neurosurgery 15, no. 4 (1984): 497–501. 6333649 10.1227/00006123-198410000-00004

[41] S. K. Gudeman , J. D. Miller , and D. P. Becker , “Failure of High‐Dose Steroid Therapy to Influence Intracranial Pressure in Patients With Severe Head Injury,” Journal of Neurosurgery 51, no. 3 (1979): 301–306. 469578 10.3171/jns.1979.51.3.0301

[42] M. Á. Carrillo‐de Sauvage , L. Maatouk , I. Arnoux , et al., “Potent and Multiple Regulatory Actions of Microglial Glucocorticoid Receptors During CNS Inflammation,” Cell Death and Differentiation 20, no. 11 (2013): 1546–1557. 24013726 10.1038/cdd.2013.108 PMC3792430

[43] K. Picard , K. Bisht , S. Poggini , et al., “Microglial‐Glucocorticoid Receptor Depletion Alters the Response of Hippocampal Microglia and Neurons in a Chronic Unpredictable Mild Stress Paradigm in Female Mice,” Brain, Behavior, and Immunity 97 (2021): 423–439. 34343616 10.1016/j.bbi.2021.07.022

[44] M. Radpour , B. Khoshkroodian , T. Asgari , H. G. Pourbadie , and M. Sayyah , “Interleukin 4 Reduces Brain Hyperexcitability After Traumatic Injury by Downregulating TNF‐α, Upregulating IL‐10/TGF‐β, and Potential Directing Macrophage/Microglia to the M2 Anti‐Inflammatory Phenotype,” Inflammation 46, no. 5 (2023): 1810–1831. 37259014 10.1007/s10753-023-01843-0

[45] X. Chen , J. Yao , J. Lai , et al., “ADAM17 Aggravates the Inflammatory Response by Modulating Microglia Polarization Through the TGF‐β1/Smad Pathway Following Experimental Traumatic Brain Injury,” Journal of Neurotrauma 40, no. 13–14 (2023): 1495–1509. 37029898 10.1089/neu.2022.0373

[46] T. Li , L. Li , R. Peng , et al., “Abrocitinib Attenuates Microglia‐Mediated Neuroinflammation After Traumatic Brain Injury via Inhibiting the JAK1/STAT1/NF‐κB Pathway,” Cells 11, no. 22 (2022): 3588. 36429017 10.3390/cells11223588 PMC9688110

[47] J. Lu , J. Wang , L. Yu , et al., “Shaoyao‐Gancao Decoction Promoted Microglia M2 Polarization via the IL‐13‐Mediated JAK2/STAT6 Pathway to Alleviate Cerebral Ischemia‐Reperfusion Injury,” Mediators of Inflammation 2022 (2022): 1707122. 35757105 10.1155/2022/1707122 PMC9232306

[48] A. Pearson , M. Koprivica , M. Eisenbaum , et al., “PPARγ Activation Ameliorates Cognitive Impairment and Chronic Microglial Activation in the Aftermath of r‐mTBI,” Journal of Neuroinflammation 21, no. 1 (2024): 194. 39097742 10.1186/s12974-024-03173-w PMC11297749

[49] W. Cai , T. Yang , H. Liu , et al., “Peroxisome Proliferator‐Activated Receptor γ (PPARγ): A Master Gatekeeper in CNS Injury and Repair,” Progress in Neurobiology 163‐164 (2018): 27–58. 10.1016/j.pneurobio.2017.10.002 PMC6037317 29032144

[50] A. Salehi , J. H. Zhang , and A. Obenaus , “Response of the Cerebral Vasculature Following Traumatic Brain Injury,” Journal of Cerebral Blood Flow and Metabolism 37, no. 7 (2017): 2320–2339. 28378621 10.1177/0271678X17701460 PMC5531360

[51] Z. A. Zhao , L. Yan , J. Wen , et al., “Cellular and Molecular Mechanisms in Vascular Repair After Traumatic Brain Injury: A Narrative Review,” Burns & Trauma 11 (2023): tkad033, 10.1093/burnst/tkad033. 37675267 PMC10478165

[52] H. C. Liu , C. H. Huang , M. R. Chiang , et al., “Sustained Release of Nitric Oxide‐Mediated Angiogenesis and Nerve Repair by Mussel‐Inspired Adaptable Microreservoirs for Brain Traumatic Injury Therapy,” Advanced Healthcare Materials 13, no. 25 (2024): e2302315. 37713592 10.1002/adhm.202302315

[53] X. Liu , C. Wu , Y. Zhang , et al., “Hyaluronan‐Based Hydrogel Integrating Exosomes for Traumatic Brain Injury Repair by Promoting Angiogenesis and Neurogenesis,” Carbohydrate Polymers 306 (2023): 120578. 36746568 10.1016/j.carbpol.2023.120578

[54] P. Chaudhari , A. Madaan , J. C. Rivera , et al., “Neuronal GPR81 Regulates Developmental Brain Angiogenesis and Promotes Brain Recovery After a Hypoxic Ischemic Insult,” Journal of Cerebral Blood Flow and Metabolism 42, no. 7 (2022): 1294–1308. 35107038 10.1177/0271678X221077499 PMC9207492

[55] N. Ferrara , “Vascular Endothelial Growth Factor,” Arteriosclerosis, Thrombosis, and Vascular Biology 29, no. 6 (2009): 789–791. 19164810 10.1161/ATVBAHA.108.179663

[56] J. A. Nagy , A. M. Dvorak , and H. F. Dvorak , “VEGF‐A and the Induction of Pathological Angiogenesis,” Annual Review of Pathology 2 (2007): 251–275. 10.1146/annurev.pathol.2.010506.134925 18039100

[57] C. Feng , Q. Tian , X. Tang , et al., “microRNA‐9a‐5p Disrupts the ELAVL1/VEGF Axis to Alleviate Traumatic Brain Injury,” Experimental Neurology 375 (2024): 114721. 38342180 10.1016/j.expneurol.2024.114721

[58] A. T. Argaw , B. T. Gurfein , Y. Zhang , A. Zameer , and G. R. John , “VEGF‐Mediated Disruption of Endothelial CLN‐5 Promotes Blood‐Brain Barrier Breakdown,” Proceedings of the National Academy of Sciences of the United States of America 106, no. 6 (2009): 1977–1982. 19174516 10.1073/pnas.0808698106 PMC2644149

[59] W. Gao , Z. Zhao , G. Yu , et al., “VEGI Attenuates the Inflammatory Injury and Disruption of Blood‐Brain Barrier Partly by Suppressing the TLR4/NF‐κB Signaling Pathway in Experimental Traumatic Brain Injury,” Brain Research 1622 (2015): 230–239. 26080076 10.1016/j.brainres.2015.04.035

[60] M. Khan , H. Khan , I. Singh , and A. K. Singh , “Hypoxia Inducible Factor‐1 Alpha Stabilization for Regenerative Therapy in Traumatic Brain Injury,” Neural Regeneration Research 12, no. 5 (2017): 696–701. 28616019 10.4103/1673-5374.206632 PMC5461600

[61] W. Yu , H. Jin , W. Sun , et al., “Connexin43 Promotes Angiogenesis Through Activating the HIF‐1α/VEGF Signaling Pathway Under Chronic Cerebral Hypoperfusion,” Journal of Cerebral Blood Flow and Metabolism 41, no. 10 (2021): 2656–2675. 33899559 10.1177/0271678X211010354 PMC8504949

[62] Z. Pan , G. Ma , L. Kong , and G. Du , “Hypoxia‐Inducible Factor‐1: Regulatory Mechanisms and Drug Development in Stroke,” Pharmacological Research 170 (2021): 105742. 34182129 10.1016/j.phrs.2021.105742

[63] Q. L. Zhang , B. R. Cui , H. Y. Li , et al., “MAPK and PI3K Pathways Regulate Hypoxia‐Induced Atrial Natriuretic Peptide Secretion by Controlling HIF‐1 Alpha Expression in Beating Rabbit Atria,” Biochemical and Biophysical Research Communications 438, no. 3 (2013): 507–512. 23916614 10.1016/j.bbrc.2013.07.106

[64] Z. Zhang , L. Yao , J. Yang , Z. Wang , and G. Du , “PI3K/Akt and HIF‐1 Signaling Pathway in Hypoxia‐Ischemia (Review),” Molecular Medicine Reports 18, no. 4 (2018): 3547–3554. 30106145 10.3892/mmr.2018.9375 PMC6131612

