# Supplementary material for: Balancing Anti‐Inflammation and Neurorepair: The Role of Mineralocorticoid Receptor in Regulating Microglial Phenotype Switching After Traumatic Brain Injury
Source: CNS Neurosci Ther. 2025 Apr 25;31(4):e70404. doi: 10.1111/cns.70404 (PMC12023002; doi:10.1111/cns.70404)
Supplement: Supplementary file 1 — Figure S1. [file CNS-31-e70404-s001.docx]

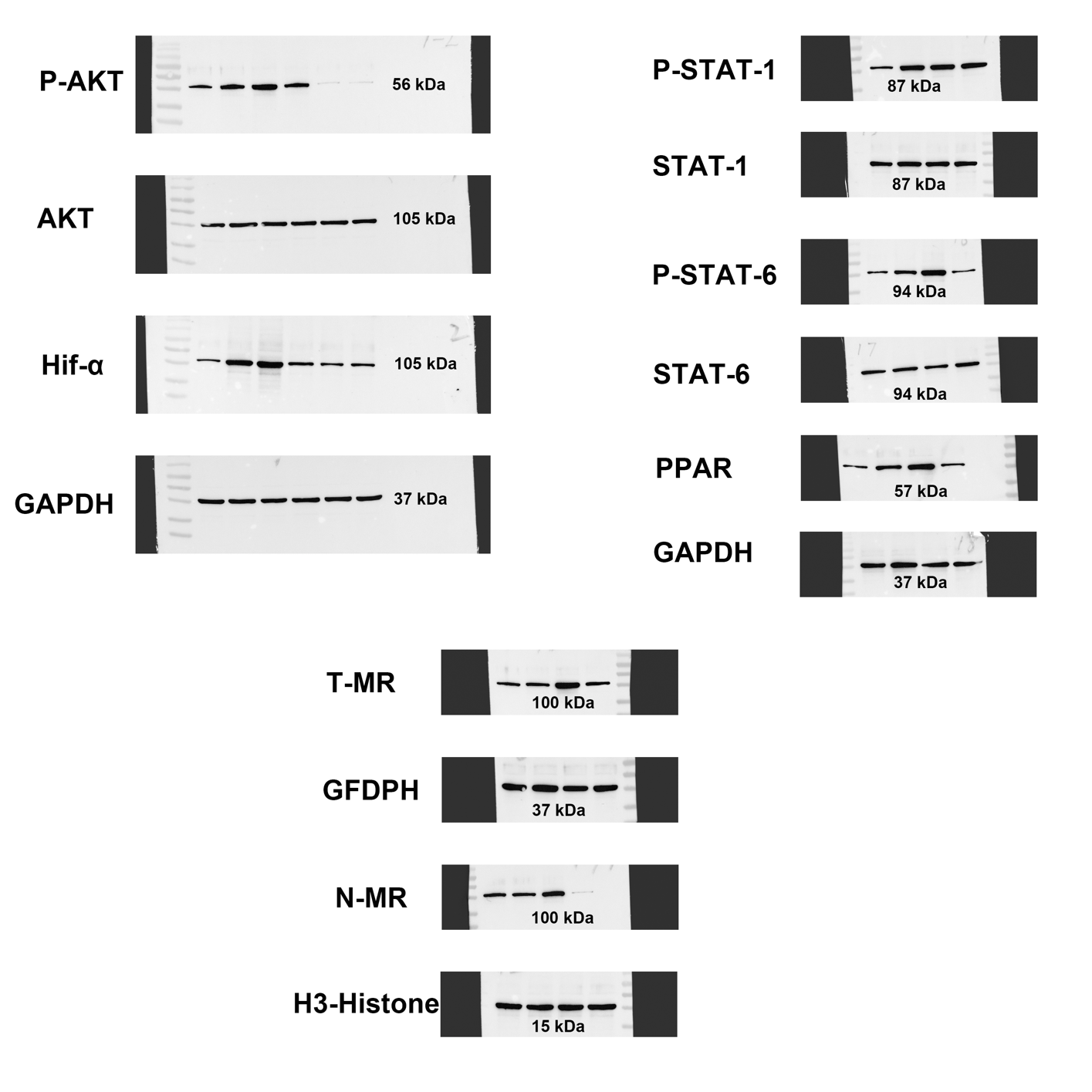


**Fig. S1.** Full uncropped scans of western blots images in Figure 8 for P-STAT1, STAT1, P-STAT6, STAT6, PPARγ, nuclear MR, total MR, GFDPH and H3-Histone; and Figure 9 for Akt, p-Akt, Hif-1α and GFDPH in the ipsilateral hemisphere.
